# Coordination‐Induced Magnetism Strategy for Highly Selective and Efficient Uranium Separation

**DOI:** 10.1002/advs.202408642

**Published:** 2024-11-04

**Authors:** Shilei Zhao, Tiantian Feng, Jiacheng Zhang, Meng Cao, Lijuan Feng, Yue Ma, Tao Liu, Yihui Yuan, Ning Wang

**Affiliations:** ^1^ State Key Laboratory of Marine Resource Utilization in South China Sea Hainan University Haikou 570228 P. R. China

**Keywords:** cyanoferrocene, magnetism conversion, separation, uranium

## Abstract

Highly efficient separation of dispersed uranium is important for the sustainable development of nuclear industry, and adsorption is the most recognized approach. However, there are many coexisting interfering metal ions that compete with uranyl ion for the chelating ligands in the adsorbents and lead to low separation selectivity and efficiency. Herein, a coordination‐induced magnetism strategy is presented for the separation of uranium based on the conversion of diamagnetic cyanoferrocene (Fc‐CN) nanocrystals to uranium‐containing magnetic recoverable ferromagnetic aggregates. Different from previous adsorption strategies, this strategy combines the mechanisms of photocatalytic uranium enrichment and chemical uranium adsorption. Under light irradiation, electron of Fe(II) in Fc‐CN is excited and transfers to uranyl ion via the cyano group to form tight coordination bond between N atom in cyano group and uranium. This phenomenon is unique for uranyl ion, and thus, a high uranium removal rate of 97.98% is achieved in simulated nuclear wastewater with the presence of tremendous interfering ions, proving its highly selective and efficient uranium separation performance. The ability to form highly stable magnetic aggregates via photoinduced interaction between Fc‐CN and uranium enriches the understanding on the chemical properties of Fc‐CN and uranium.

## Graphical abstract

This study proposes a coordination‐induced magnetism strategy for the uranium separation based on the conversion of diamagnetic cyanoferrocene (Fc‐CN) nanocrystals into magnetically recoverable uranium‐containing ferromagnetic aggregates. This strategy achieves a high uranium removal rate of 97.98% in simulated nuclear wastewater in the presence of a large number of interfering ions, proving the application potential of this uranium separation strategy.

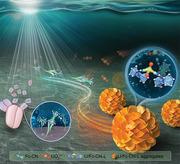

## 1 Introduction

Uranium is a key resource for the sustainable development of nuclear industry, which is crucial for achieving the strategic goal of carbon neutrality.^[^ ^1^ ^]^ Based on current consumption rate, terrestrial uranium reserves can last for only 60–80 years, making it an urgent need to explore new sources of uranium.^[^ ^2^ ^]^ Furthermore, uranium exhibits chemotoxicity and radioactive toxicity.^[^ ^1^, ^3^ ^]^ The mining of terrestrial uranium ores, improper disposal of uranium‐containing waste, and the use of depleted uranium bombs for military applications have caused environmental uranium pollution, which subsequently poses hazards to environmental security and human health.^[^ ^4^ ^]^ The recovery of dispersed uranium can contribute to the sustainable access of uranium resources and reduces the harmful environmental impacts of uranium pollution.^[^ ^5^ ^]^ Adsorption is considered the most promising strategy for the separation of dispersed uranium.^[^ ^6^ ^]^ However, in natural environment, numerous metal ions coexist and compete with uranyl ions, the dominant form of uranium in the environment, for chelating ligands in the adsorbents.^[^ ^7^ ^]^ Catalytic enrichment approaches have emerged as a new strategy for recovering uranium resources. Nevertheless, the catalysts mainly exist in the form of powders and are difficult to recover after being launched into aqueous environments. Thus, the recovery of dispersed uranium from complicated aqueous conditions remains a challenge in terms of efficiency, speed, and selectivity of available uranium separation approaches.

Magnetic separation is a promising strategy for recovering target substances from complex conditions and has been commercially applied in numerous areas. In combination with chelating ligands, the magnetic separation strategy exhibits the advantages of high separation selectivity, fast separation speed, easy operation, and excellent separation efficiency. Magnetic separation materials have been applied for the recovery of dispersed uranium.^[^ ^8^ ^]^ However, the utilization of traditional chelating ligands along with these magnetic uranium separation materials has led to low recovery efficiency.^[^ ^9^ ^]^ Owing to the large radial extension and strong spin–orbit‐coupling of uranium, its *5f* orbitals are found to overlap with the *3d* orbitals of transition metal ions,^[^ ^10^ ^]^ resulting in a magnetic coupling effect for fabricating molecular magnetic materials.^[^ ^11^ ^]^ This unique magnetic conversion feature of uranium can differentiate uranyl ions from other coexisting ions and, in principle, can address the challenges pertaining to the separation of uranium from a complicated environment by enhancing separation selectivity. To date, uranium‐induced magnetic conversion properties have only been merely investigated, and have never been used for selective uranium separation.

**Figure 1:**
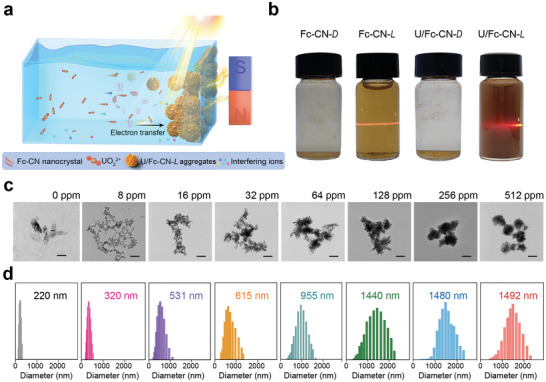
Schematic illustration and characteristics of the assembly of Fc‐CN nanocrystals with the induction of light irradiation and uranyl ions. a) Schematic illustration of the assembly of Fc‐CN nanocrystals into magnetic aggregates for magnetic separation of uranium from a complicated environment. b) Transformation of Fc‐CN under different conditions in an aqueous environment by the change of the Tyndall effect, c) particle morphology, and d) particle size. The concentrations of uranyl ions are expressed as the concentration of uranium. The average sizes of the particles are also indicated. Scale bars are 200 nm.

Ferrocene (Fc) is composed of cyclopentadiene and *3d* transition metal Fe.^[^ ^12^ ^]^ With external stimulation, including light and heat, Fe(II) in Fc and its derivatives are prone to function as electron donors, thereby leading to a change in the electron state and facilitating the generation of magnetism.^[^ ^13^ ^]^ Cyanoferrocene (Fc‐CN) is a derivative of Fc, and nanocrystals of Fc‐CN exhibit diamagnetic character. Herein, to realize highly efficient recovery of dispersed uranium, Fc‐CN nanocrystals are applied. Under light irradiation, the N atom in the C≡N group of Fc‐CN is found to coordinate with the uranium atom in uranyl ion to mediate the assembly of the Fc‐CN nanocrystals into micrometer aggregates, named as U/Fc‐CN‐*L* (**Figure** **1**a). This coordination between uranyl ions and the C≡N group in Fc‐CN causes changes in the electron spinning state of Fe atom, which empowers the formed micrometer aggregates with room temperature magnetism that can be utilized for the magnetic separation of dispersed uranium from complicated aqueous environments. The photo‐induced magnetically converted behavior is only observed for the uranyl ion, but not for the other metal ions, which ensures the high separation selectivity of this strategy. The finding of this study presents a novel uranium‐induced magnetically converted strategy for highly selective and efficient separation of dispersed uranium, as well as enriches the understanding on the chemical properties of uranium.

## 2 Results and Discussion

### 2.1 Essential Factors for Forming Magnetic U/Fc‐CN‐*L* Aggregates

Nanocrystals of Fc‐CN were fabricated through the recrystallization of Fc‐CN in a mixture of tetrahydrofuran and deionized water. The initial Fc‐CN nanocrystals exhibit a sheet‐like structure with an average particle size of 220 nm (Figure S1, Supporting Information). The crystal structure and chemical composition of the fabricated nanocrystals correspond to the theoretical characteristics of the Fc‐CN crystal (Figure S2, Tables S1 and S2, Supporting Information). To determine the essential factors for inducing the assembly of Fc‐CN nanocrystals to form magnetic aggregates, the effects of light irradiation and uranyl ions on the assembly behavior were investigated. The initial Fc‐CN nanocrystals exhibit a diamagnetic character and cannot disperse in water under dark conditions, which exist as precipitates, designated as Fc‐CN‐*D* (Figures S3 and S4, Supporting Information). Upon light irradiation, the Fc‐CN nanocrystals transform into uniformly dispersed thinner nanosheets in water, designated as Fc‐CN‐*L*, and exist in the form of colloids due to the appearance of the Tyndall effect (Figure 1b; and Movie S1 and Figure S5, Supporting Information). Furthermore, the presence of both light irradiation and uranyl ions in the aqueous mixture induces the assembly of Fc‐CN nanocrystals into aggregates, designated as U/Fc‐CN‐*L*, with an increased particle size and show nanoflower‐like morphology (Figure 1c; and Figure S6, Supporting Information). Uranyl ions alone cannot induce the assembly of Fc‐CN nanocrystals into aggregates without light irradiation, indicating that both light irradiation and uranyl ions are essential for the assembly of Fc‐CN nanocrystals into U/Fc‐CN‐*L* aggregates (Figure S7, Supporting Information). The magnetic behaviors of Fc and ferrocenecarboxylic acid (Fc‐COOH) are also investigated upon uranium exposure and light irradiation. Both of the treated derivatives, referred to as U/Fc‐*L* and U/Fc‐COOH‐*L*, exhibit negligible saturation magnetizations (Ms), highlighting the crucial role of the cyano group in inducing the magnetism of the Fc‐CN nanocrystals (Figure S8, Supporting Information). An increase in the uranyl ion concentration results in the formation of U/Fc‐CN‐*L* aggregates with larger particle sizes. Owing to the increase in the particle size of the aggregates, the Tyndall effect of U/Fc‐CN‐*L* formed under high uranyl ion concentrations decreases. Dynamic light scattering analysis confirms the assembly of Fc‐CN under light irradiation in the presence of uranyl ions, and the particle size of the aggregates is positively correlated with the concentration of uranyl ions (Figure 1d).

**Figure 2:**
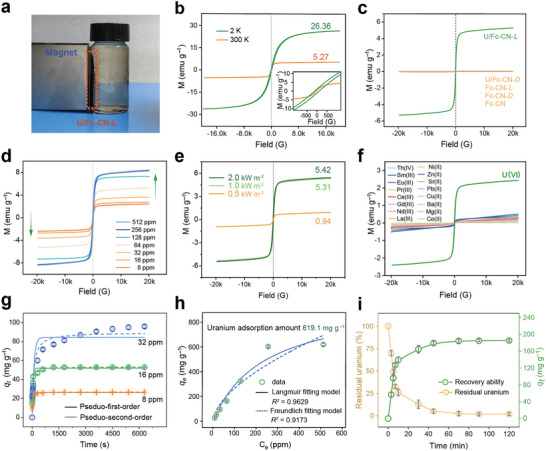
Factors for inducing the generation of magnetic U/Fc‐CN‐*L* aggregates to separate uranium by Fc‐CN nanocrystals. a) Magnetic separation ability of U/Fc‐CN‐*L*. b) Magnetization curves of U/Fc‐CN‐*L* at 2 K and 300 K. U/Fc‐CN‐*L* is fabricated with an initial uranium concentration of 64 ppm. c) Magnetization curves of Fc‐CN nanocrystals with different treatments. d) Magnetization curves of U/Fc‐CN‐*L* fabricated with different concentrations of uranyl ions. e) VSM of U/Fc‐CN‐*L* prepared using the uranyl ion solution with a uranium concentration of 64 ppm under different light intensities. f) Magnetization curves of Fc‐CN nanocrystals treated using different metal ions under light irradiation (Uranium: 8 ppm; other metal ions: 40 ppm). g) Separation kinetics of uranium from a uranyl solution by Fc‐CN nanocrystals (0.3 g L^−1^) at pH 5.0 under 1 kW m^−2^ light irradiation at 298.15 K. h) Separation of uranium from uranyl solutions with different concentrations of uranium by Fc‐CN nanocrystals (0.3 g L^−1^) at pH 5.0 and 298.15 K. i) Separation kinetics of uranium from simulated nuclear wastewater at pH 5.0 with initial uranium concentration of 18.9 ppm by Fc‐CN nanocrystals (0.1 g L^−1^) at 298.15 K. The data are presented as mean ± standard deviation (SD).

The magnetism of the Fc‐CN nanocrystals subjected to different treatments was analyzed. Results show that the formed U/Fc‐CN‐*L* aggregates exhibit magnetic response behavior and can be easily recovered by using a magnet with a low magnetic field strength of 0.3 T (**Figure** **2**a; and Movie S2, Supporting Information). However, the initial Fc‐CN nanocrystals, Fc‐CN‐*D*, Fc‐CN‐*L*, and U/Fc‐CN‐*D*, lack the ability to be attracted by magnets (Figure S9, Supporting Information). A vibrating sample magnetometer (VSM) was used to analyze the magnetic characteristics in detail. Results show that U/Fc‐CN‐*L* exhibits a low coercive force at 300 K and a higher coercive force at 2 K (Figure 2b). Furthermore, the saturation magnetization of U/Fc‐CN‐*L* increases from 5.27 emu g^−1^ at 300 K to 26.36 emu g^−1^ at 2 K. The zero‐field cooling and field cooling curves of U/Fc‐CN‐*L* under an applied magnetic field with a strength of 100 Oe together with the results of the VSM analysis prove the nature of U/Fc‐CN‐*L* as a ferromagnet (Figure S10, Supporting Information). These results further demonstrate that both light irradiation and uranyl ions are essential for the generation of magnetism (Figure 2c). U/Fc‐CN‐*L* exhibits an increased Ms with increasing uranyl ion concentrations or light intensities for fabricating U/Fc‐CN‐*L*, which corresponds with the assembly behavior of Fc‐CN under these conditions (Figure 2d,e; and Figure S11 Supporting Information). Compared with that of U/Fc‐CN‐*L* with a maximum particle size of 1492 nm, the Fc‐CN crystal with a bigger particle size of 76 µm still lacks the magnetic response behavior, further confirming that Fc‐CN nanocrystals undergo a physical property transformation with the induction of light irradiation and uranyl ions for the generation of magnetism (Figure S12, Supporting Information). The magnetic transformation of Fc‐CN nanocrystals is found to be specific for uranyl ions, as the other 16 tested metal ions, including Th(IV), Sm(III), Eu(III), Pr(III), Ce(III), Gd(III), Nd(III), La(III), Ni(II), Zn(II), Sr(II), Pb(II), Cu(II), Ba(II), Mg(II), and Co(II), cannot induce the magnetic response behavior under light irradiation, which can benefit the selective separation of uranium from a complicated environment containing diverse coexisting metal ions (Figure 2f).

### 2.2 Magnetic Separation of Uranium via Forming U/Fc‐CN‐*L* Aggregates

Based on the ability to produce magnetically recyclable U/Fc‐CN‐*L*, the magnetic separation ability of the Fc‐CN nanocrystals for uranium was evaluated. Results show that similar to the increase in magnetic response behavior, an increase in light intensity leads to an increase in uranium separation ability, and a uranium separation rate of ≈100% can be achieved with a light intensity of 1 kW m^−2^ (Figure S13, Supporting Information). Even under natural sunlight with a light intensity of 0.93–0.97 kW m^−2^, Fc‐CN nanocrystals can still remove more than 80% uranium from the uranyl ion solution (Figure S14, Supporting Information). Moreover, the uranium separation rate of Fc‐CN nanocrystals is significantly higher than that of Fc, confirming the crucial role of the cyano group for uranium separation (Figure S15, Supporting Information).^[^ ^14^ ^]^ The optimal dosage of Fc‐CN nanocrystals for uranium recovery has also been determined, and the maximum equilibrium separation rate of 99.4% can be achieved by Fc‐CN nanocrystals with a dosage of 0.3 g L^−1^ (Figure S16, Supporting Information). The uranium separation performance of Fc‐CN nanocrystals is further evaluated across a pH range from 3 to 8 and the optimal pH for uranium separation is determined to be pH 5 (Figure S17, Supporting Information). In addition, the uranium separation capacity of Fc‐CN is evaluated in uranium solutions with concentrations of 8, 16, and 32 ppm. The adsorption kinetics can be well described by the pseudosecond order model, suggesting the dominance of chemisorption (Figure 2g; and Figure S18 and Table S3, Supporting Information). The adsorption isotherms fit with the Langmuir model (*R* ^2^ = 0.9629) better than the Freundlich model, indicating the monolayer adsorption (Figure 2h). The maximum adsorption capacity reaches 619.1 mg g^−1^ in 512 ppm uranium solution, closely approaching the theoretical maximum adsorption capacity of 663.2 mg g^−1^. It is important to note that only a short equilibrium period of less than 20 min is needed to remove more than 90% of the uranium from a solution with a uranium concentration lower than 16 ppm, which can benefit the practical application of Fc‐CN nanocrystals for rapid uranium recovery. Furthermore, free energy of adsorption (Δ*G*_ads_) is calculated based on the Langmuir isotherm, and the obtained Δ*G*_ads_ is negative (−0.475 kJ mol^−1^), indicating that the adsorption of uranium by Fc‐CN nanocrystals is spontaneous under light irradiation. Owing to the specific interaction mechanism between Fc‐CN and uranyl ions, the Fc‐CN nanocrystals show a highly selective uranium separation ability, which avoids the interference of coexisting metal ions (Figure S19, Supporting Information).

The practical application potential of Fc‐CN nanocrystals was determined in high‐salinity simulated nuclear wastewater with initial uranium concentration of 18.9 ppm. In dark condition, Fc‐CN nanocrystals with a dosage of 0.1 g L^−1^, demonstrate a uranium enrichment performance of 16.77 mg g^−1^ (Figure S20, Supporting Information). However, under light conditions, Fc‐CN nanocrystals removed 97.98% of uranium and achieved a high enrichment performance of 185.18 mg g^−1^ within 70 min, which is ≈11 times higher than that under dark condition (Figure 2i). Therefore, Fc‐CN nanocrystals are among the best performing adsorbents for the recovery of uranium from nuclear wastewater, outperforming traditional Fe_3_O_4_‐based materials in both uranium adsorption capacity and adsorption rate (Tables S4–S6, Supporting Information). Owing to the high separation selectivity of Fc‐CN nanocrystals, their uranium‐separation efficiency is unaffected by the presence of multiple interfering cations, and the presence of diverse anions only slightly affects their uranium‐separation performance (Figures S21 and S22, Supporting Information). These excellent properties make the Fc‐CN nanocrystal a promising candidate for practical uranium separation in complicated environments.

### 2.3 Mechanism for Forming Magnetic U/Fc‐CN‐*L* Aggregates

**Figure 3:**
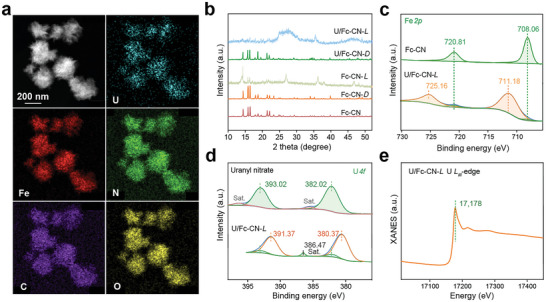
Chemical characteristics of U/Fc‐CN‐*L*. a) TEM and corresponding EDX analysis of U/Fc‐CN‐*L*. b) Powder X‐ray diffraction (PXRD) spectra of Fc‐CN nanocrystals undergoing different treatments. c) High‐resolution Fe *2p* XPS spectra of Fc‐CN and U/Fc‐CN‐*L*_._ d) High‐resolution U *4f* XPS spectra of uranyl nitrate and U/Fc‐CN‐*L*_._ e) U *L*_III_‐edge XANES spectrum of U/Fc‐CN‐*L*.

The chemical properties of U/Fc‐CN‐*L* were analyzed in detail to uncover its formation mechanism. Energy‐dispersive X‐ray (EDX) analysis confirms the presence of C, N, O, Fe, and U elements in U/Fc‐CN‐*L* (**Figure** **3**a). Fourier transform infrared (FTIR) analysis also proves the binding of uranyl ions, as the appears of the characteristic peak for uranyl ion at 926 cm^−1^ (Figure S23, Supporting Information). Furthermore, the intensities of peaks for C−H and C═C groups increase after the binding of uranyl ions by Fc‐CN nanocrystals because of the polarity alteration caused by the interaction between Fc‐CN and uranyl ions.^[^ ^15^ ^]^ The thermogravimetric analysis (TGA) shows that at temperatures higher than 470 °C, the masses of U/Fc‐CN‐*L* and Fc‐CN are reduced to 50.45% and 10% of the initial mass, respectively, indicating that the bound uranyl ions take up ≈40.45% of the mass (Figure S24, Supporting Information). The formed U/Fc‐CN‐*L* exhibits high stability and can resist 2 m HNO_3_, 2 m HCl, 2 m NaOH, and different organic solvents (Figure S25, Supporting Information). Considering the high stability and magnetism of the formed aggregates, as well as the simple fabrication process, this method of preparing U/Fc‐CN‐*L* can also be utilized for the fabrication of other new magnetic materials.

After interacting with uranyl ions, Fc‐CN exhibits a significant change from crystalline structure to amorphous structure, which is attributed to the recombination of Fc‐CN molecules during the interaction with uranyl ions (Figure 3b). Compared with the initial Fc‐CN nanocrystals, Fc‐CN‐*L* shows reduced crystallinity, suggesting that light can drive the recombination of molecules in the Fc‐CN nanocrystals, thereby increasing the interaction ability between Fc‐CN and uranyl ions in aqueous environment. X‐ray photoelectron spectroscopic (XPS) analysis shows that the C−C bond binding energy is calibrated to be 284.80 eV (Figure S26, Supporting Information). The Fe *2d* spectra reveals that the binding energy of Fe is increased after the formation of U/Fc‐CN‐*L* from Fc‐CN, while the binding energy of U is reduced, which is due to the transfer of electrons from Fe(II) to U(VI) (Figure 3c,d). In addition, the binding energy difference between the U *4f*_7/2_ satellite peak and the U *4f*_7/2_ main peak in U/Fc‐CN‐*L* is about 6.1 eV, which is consistent with the difference in the characteristic binding energy of U(IV), confirming the presence of U(IV) species in U/Fc‐CN‐*L*.^[^ ^16^ ^]^ The electron transfer process is achieved through the formation of the N−U bonds in U/Fc‐CN‐*L* (Figure S27, Supporting Information). The valence states of Fe and U in U/Fc‐CN‐*L* were confirmed using X‐ray absorption near‐edge structure (XANES) analysis. The U *L*_III_‐edge adsorption energy of U/Fc‐CN‐*L* is determined to be 17178 eV, which is close to that of UO_2_ species at 17177.2 eV (Figure 3e).^[^ ^17^ ^]^ In addition, Fe in U/Fc‐CN‐*L* shows an adsorption energy similar to that of Fe in Fe_2_O_3_ (Figure S28, Supporting Information). These results show that electron transfer from Fe(II) to U(VI) occurs during the photoinduced interaction between Fc‐CN and uranyl ions.

**Figure 4:**
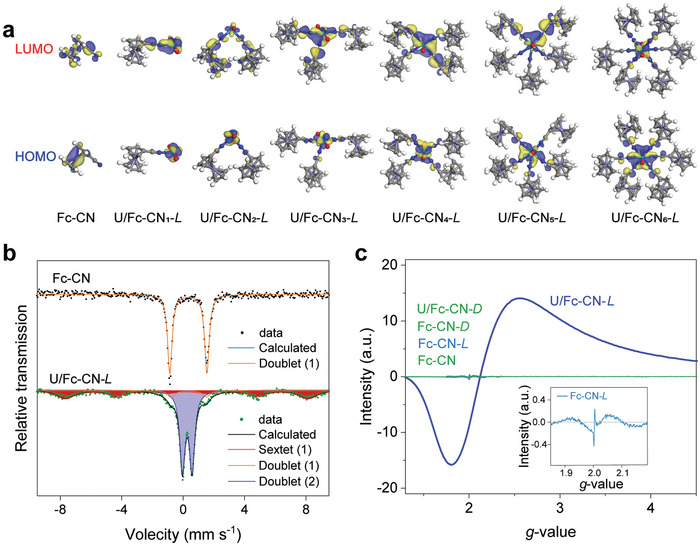
Determination of the magnetic generation mechanism. a) HOMO‐LUMO diagrams of U/Fc‐CN*_n_*‐*L* (*n* = 1–6). b) Room‐temperature ^57^Fe Mössbauer spectra of Fc‐CN and U/Fc‐CN‐*L*. c) EPR spectra of Fc‐CN with different treatments.

The mechanism of interaction between Fc‐CN and uranyl ions for generating magnetic U/Fc‐CN‐*L* aggregates was analyzed. Optical and electronic analyses of the Fc‐CN nanocrystals show that the electrons in Fc‐CN can be excited under light irradiation (Figures S28–S31, Supporting Information). Results of the Mott–Schottky test show that the flat band (*E*_fb_) potential of Fc‐CN is −0.289 V (vs Ag/AgCl electrode), corresponding to −0.089 V (vs normal hydrogen electrode), which is lower than the redox potentials of UO_2_^2+^/U^4+^ (0.267 V) and UO_2_^2+^/UO_2_ (0.411 V), confirming the electron transfer ability from Fc‐CN to uranyl ions under light irradiation (Figure S32, Supporting Information). For density functional theory (DFT) calculations, six different binding models of uranyl ions by Fc‐CN molecules were analyzed by changing the ratio of Fc‐CN to uranyl ions in U/Fc‐CN*_n_*‐*L* (*n* = 1–6). Results of generated density of states reveal an overlap between the N *p* orbit and the U *f* orbit, suggesting that the hybridization between the orbits of the N and U atoms is possible to be the interaction mechanism between Fc‐CN and uranyl ions (Figures S33 and S34, Supporting Information). The frontier molecular orbital theory was further applied to predict the electronic distribution and electron transport pathways from Fc‐CN to the uranyl ion. Results show that, in Fc‐CN, the highest occupied molecular orbital (HOMO) is mainly distributed on the Fe atom and the lowest unoccupied molecular orbital (LUMO) is mainly distributed on the C≡N group, indicating the electron transfer from the Fe atom to C≡N (**Figure** **4**a). However, after the binding of uranyl ions, the LUMOs of U/Fc‐CN*_n_*‐*L* are all distributed on the uranium atom, indicating that electrons can further transfer from the C≡N group to the uranium atom. The coordination environment of uranium in the U/Fc‐CN‐*L* was further investigated using EXAFS spectroscopy analyses with uranium concentration of 512 ppm (Figure S35 and Table S7, Supporting Information). The EXAFS result analysis revealed that in U/Fc‐CN‐*L*, the central uranium atom is coordinated with two axial oxygen atoms at a bond length of 1.97 Å, four equatorial oxygen atoms at a bond length of 2.21 Å, and two equatorial nitrogen atoms at a bond length of 2.39 Å. The coordinated four equatorial oxygen atoms come from water molecules in the solution, and the coordinated nitrogen atoms come from the −CN of Fc‐CN.

Results of ^57^Fe Mössbauer spectroscopic analysis show that, based on the values of isomer shift and quadrupole splitting, the Fe in Fc‐CN exhibits one doublet (D1) with a peak area of 100%, which is corresponding to the high spin (HS) Fe(II) (Figure 4b; and Figure S36 and Table S8, Supporting Information). However, the Fe in U/Fc‐CN‐*L* shows three different types of peaks, including two doublet peaks (D1 and D2) corresponding to HS Fe(II) and HS Fe(III), respectively, and a sextet peak corresponding to HS Fe(III). Notably, the areas of the HS Fe(II) peaks for Fc‐CN decrease significantly from 100% to 6.3% for U/Fc‐CN‐*L*, indicating the conversion of HS Fe(II) to HS Fe(III). Electron paramagnetic resonance (EPR) spectroscopy analysis shows the appearance of a signal peak that corresponds to the unpaired spin electrons of Fe(III), which is absent for Fc‐CN, confirming the conversion of Fe(II) in Fc‐CN to Fe(III) in U/Fc‐CN‐*L* (Figure 4c). The conversion of Fe(II) to Fe(III) causes a reduction in the total number of spin electrons in U/Fc‐CN‐*L* and an increase in the number of unpaired spin electrons, resulting in an increase in the molecular magnetic moment, thereby making U/Fc‐CN‐*L* magnetic (Figure S37, Supporting Information).

## 3 Conclusion

Separation of dispersed uranium resource is important for fueling the nuclear energy industry and for protecting human from the hazards of environmental uranium pollution. The adsorption strategies are widely studied for the separation of dispersed uranium resources. However, they suffer from the shortages of low separation selectivity and difficult for practical application. Thus, the exploration of new principles for uranium separation is urgent needed. This study for the first time discovers the phenomenon that uranyl ions can induce the magnetic conversation of diamagnetic Fc‐CN nanocrystals into magnetic‐separable ferromagnetic aggregates under light irradiation. The mechanisms for the binding of uranyl ions by Fc‐CN and the magnetic conversation of diamagnetic Fc‐CN nanocrystals into ferromagnetic aggregates are uncovered. Based on this phenomenon and the corresponding mechanism, a novel magnetic conversion strategy is designed for the separation of dispersed uranium resources, which can achieve the high uranium enrichment capacity of 619.1 mg g^−1^. More importantly, this strategy can remove 97.98% of uranium from simulated high‐salinity nuclear wastewater, proving the high application potential of this magnetic conversion uranium separation strategy.

## 4 Experimental Section

### Materials

Fc (98%), Fc‐CN (98%), uranyl nitrate (UO_2_(NO_3_)_2_·6H_2_O, 99%), and arsenazo III (95%) were purchased from Shanghai Macklin Biochemical Technology Co., Ltd. Nitric acid (HNO_3_, 70%) was purchased from Thermo Fisher Technology Co., Ltd. Sodium hydroxide (NaOH, 98.5%) was obtained from Sigma‐Aldrich Trading Co., Ltd. To further improve the purity of Fc‐CN, it was recrystallized in a mixture of tetrahydrofuran and deionized water at a volume ratio of 2:1.

### Characterization

Scanning electron microscopy (SEM) images were obtained using a field‐emission scanning electron microscope (S‐4800, Hitachi, Japan). Transmission electron microscopy (TEM, FEI Talos 200S, Thermo Fisher Scientific, USA) and EDX were used to characterize the morphology and elemental composition. The particle sizes of the samples were determined using dynamic light scattering (Malvern Zetasizer Nano ZS90, Malvern Instruments, UK) and analyzed using Zetasizer software. Magnetic tests were performed using a VSM (PPMS‐9 system, Quantum Design, USA). The FC/ZFC magnetization curve was obtained using a superconducting quantum interference device magnetometer (MPMS3, Quantum Design, USA) with external magnetic fields of 0.01 T between the temperatures of 2 and 300 K. EPR (A300‐10/12, Bruker, Germany) was used to detect the uncoupled electrons. The elemental electron binding energies were determined using XPS (ESCALAB 250XI, Thermo Electron Corporation, USA). The spectra were deconvoluted using the fitting program XPSPEAK 4.1. XANES spectra were analyzed using the BL14W1 beamline of the Shanghai Synchrotron Radiation Facility and the signal was detected using a Lytle detector (BL14W1, SSRF, China). UV–Visible diffuse reflection spectroscopy was performed using a UV–Vis spectrophotometer (UV‐3600i PLUS, Shimadzu, Japan). Photoluminescence (PL) spectra were measured using an Edinburgh (UK) FLS1000 spectrophotometer at 298.15 K. Infrared adsorption spectra were collected using a FTIR spectrometer (LR‐64912C, PerkinElmer, USA). The crystal structure of the recrystallized Fc‐CN was collected at 293 K on a single crystal X‐ray diffractometer (Bruker D8 Venture, Germany) using Mo Kα radiation in the 2*θ* range of 2.689°–26.364°. Single‐crystal structures of the samples were determined using the program Olex2. PXRD data were collected using a PXRD analyzer (Smartlab‐9KW, Rigaku, Japan) using Cu Kα radiation at a scanning rate of 2° min^−1^ for a 2*θ* range of 10°–55°. A nuclear magnetic resonance (Advance 400, Bruker, Germany) spectrometer was used to analyze the chemical structures of the materials. The residual contents of Fe and uranium in the U/Fc‐CN‐*L* were determined using TGA (TGA 550, TA Instruments, USA) at a heating rate of 10 °C min^−1^. An organic elemental analyzer (Elementar Vario EL, Germany) was used to analyze the elemental content of Fc‐CN. Relative molecular weight was determined using mass spectrometry (MS, U3000 LC‐20AD, Shimadzu, Japan). ^57^Fe Mössbauer spectra were recorded on a Wissel MR‐2500 Mössbauer spectrometer (Germany) using a ^57^Co (Pd) source in the transmission geometry. Data were fitted using the MossWinn software (version 4.0). The adsorption abilities of uranium and competing ions were determined using inductively coupled plasma emission spectroscopy (ICP‐OES, Agilent 5900, Agilent Technologies, USA) and a UV–Vis absorption spectrophotometer (UV‐1600, Shanghai Mapada Instrument, China).

### Electrochemical and Photoelectrochemical Measurements

The transient photocurrent responses (*i*–*t*), electrochemical impedance spectroscopy (EIS), and Mott–Schottky curves were obtained using the CHI‐660E electrochemical workstation (Chenhua, Shanghai, China) with a conventional three‐electrode configuration, including working, counter, and reference electrodes. A working electrode with an active area of 1 cm^2^ was prepared by using 10 mg of the material. A Pt plate and saturated Ag/AgCl were used as the counter and reference electrodes, respectively. EIS measurements were performed under light conditions. The frequency range was 0.1–100 KHz and the electrolyte was a 0.2 m Na_2_SO_4_ solution. Mott–Schottky measurements were carried out at a frequency of 1000 Hz with a potential range of −1–1 V.

### Magnetic Performance Experiments

Factors for the transformation of Fc‐CN nanocrystals into magnetic aggregates: Fc‐CN nanocrystals with individual weights of 6 mg were placed in four 20 mL vials to determine the key factors that lead to the formation of their magnetic aggregates. Two of these four vials were filled with 20 mL of deionized water and subjected to either dark or light irradiation. The other two vials were filled with 20 mL of uranyl nitrate solution with a uranium concentration of 64 ppm and treated with either dark or light irradiation. The treatments were performed at 298.15 K for 4 h with a light intensity of 1.0 kW m^−2^. After the treatment, the product was centrifuged at 10 000 rpm for 5 min at 298.15 K and further freeze‐dried for analysis of magnetic properties.

Effect of uranium concentrations on the magnetic performance of U/Fc‐CN‐*L*: To explore the effect of uranium concentration on the magnetic properties of U/Fc‐CN‐*L*, uranyl nitrate solutions with the highest uranium concentration of 512 ppm were used to fabricate U/Fc‐CN‐*L*. For each treatment, 6 mg of recrystallized Fc‐CN nanocrystals were added to 20 mL of uranyl nitrate solutions of different uranium concentrations. The treatments were performed at 298.15 K for 4 h with a light intensity of 1.0 kW m^−2^. The formed products were collected using centrifugation and subjected to magnetic property analysis after freeze‐drying.

Effect of light intensity on the magnetic performance of U/Fc‐CN‐*L*: To explore the effect of light intensity on the magnetic properties of U/Fc‐CN‐*L*, 6 mg of recrystallized Fc‐CN nanocrystals were added to 20 mL of uranyl nitrate solution with a uranium concentration of 64 ppm. Light with intensities of 0.5, 1.0, and 2.0 kW m^−2^ were used to irradiate the mixture. The treatments were performed for 4 h at 298.15 K. The formed products were collected using centrifugation and subjected to magnetic property analysis after freeze‐drying.

Specificity of magnetic performance of Fc‐CN to uranium: Eighteen samples of Fc‐CN nanocrystals, each weighing 6 mg, were individually added to 40 ppm solutions of 18 metal ions, including Na(I), K(I), Th(IV), Sm(III), Eu(III), Pr(III), Ce(III), Gd(III), Nd(III), La(III), Ni(II), Zn(II), Sr(II), Pb(II), Cu(II), Ba(II), Mg(II), and Co(II). After light irradiation treatment at 298.15 K for 4 h at 1.0 kW m^−2^, samples were collected using centrifugation to investigate the magnetic response performances of Fc‐CN nanocrystals to these 18 tested metal ions.

### Uranium Separation Experiments

Uranium separation kinetics of Fc‐CN nanocrystals: To determine the uranium separation capability, 6 mg of Fc‐CN nanocrystals were added to 20 mL of uranium solution (*C*_0_ = 8, 16, and 32 ppm) and irradiated at 298.15 K for 4 h with a light intensity of 1.0 kW m^−2^. The aqueous solution was collected during the separation process and filtered using a 0.22‐µm filter to remove the suspended particles. The uranium concentration in the solution was measured using the arsenazo III spectrophotometric method to determine uranium separation kinetics. Uranyl nitrate solutions with uranium concentrations of 8, 16, 32, 64, 128, 256, and 512 ppm were used to determine the maximum equilibrium enrichment capacities of the Fc‐CN nanocrystals. For these tests, 6 mg of Fc‐CN nanocrystals was added to 20 mL of uranyl nitrate solutions with different uranium concentrations. The sorption efficiency (*SE*) was calculated using Equation (1)^[^ ^18^ ^]^

(1) $$\mathrm{SE}\;\left(\%\right)=\frac{C_{0}-C_{t}}{C_{0}}\;\times 100\%$$

The uranium enrichment capacity (mg g^−1^) *q*_t_ was calculated using Equation (2)^[^ ^19^ ^]^

(2) $$q_{t}=\frac{\left(C_{0}-C_{e}\right)V}{m}$$

where *C*_0_ (ppm) is the initial uranium concentration, *C*_t_ (ppm) is the uranium concentration at enrichment time t, *C*_e_ (ppm) is the equilibrium uranium concentration, *V* is the volume of the solution (L), and *m* is the mass of Fc‐CN nanocrystals (g).

Uranium enrichment capacity assay in simulated nuclear wastewater: Simulated nuclear wastewater was prepared to measure the uranium enrichment capacity of Fc‐CN nanocrystals (Table S4, Supporting Information).^[^ ^20^ ^]^ 5 mg of Fc‐CN nanocrystals were added to 50 mL of simulated nuclear wastewater and irradiated at 298.15 K for 4 h with light intensity of 1.0 kW m^−2^. Aqueous samples were collected during the adsorption process, the concentrations of uranium in the solutions were determined using ICP‐OES, and the separation kinetics in simulated nuclear wastewater were determined accordingly.

### Computational Details

DFT calculations were performed using Materials Studio CASTEP 2020.^[^ ^21^ ^]^ The generalized gradient approximation (GGA) method with the Perdew–Burke–Ernzerhof function was used to describe the interactions between the valence electrons and ionic core.^[^ ^22^ ^]^ The energy cutoff for the plane‐wave basis was set at 450 eV. The threshold values of the convergence criteria were specified as follows: 0.05 eV Å^−1^ for the maximum force, 0.002 Å for maximum displacement, 0.05 GPa for the maximum stress, 10^−1^ eV atom^−1^ for energy, and 2.0 × 10^6^ eV atom^−1^ for self‐consistent field tolerance. The Fc‐CN and U/Fc‐CN‐*L* were optimized within a 10 × 10 × 10 Å box. Upon completing the optimization process, the spin density of states was obtained using the GGA method. Additionally, DFT calculations were performed using the Dmol3 module of Materials Studio 2020 with the convergence criteria for force and energy set at 0.002 Ha Å^−1^ and 10^−5^ Ha, respectively. After optimization, the highest occupied molecular orbitals and lowest unoccupied molecular orbitals were determined.

### Statistical Analysis

All data processing and analysis were performed using Origin 2021 software. Results were presented as mean ± standard deviation (SD) from three independent experiments.

## Conflict of Interest

The authors declare no conflict of interest.

## Author Contributions

N.W. and Y.Y. conceived and supervised the study. Y.Y., N.W., and S.Z., conceived and designed the experiments. S.Z. and T.F. carried out all the experiments, analyzed data, and wrote the manuscript. Y.Y., N.W., S.Z., T.F., J. Z., M.C., L.F., Y.M., and T.L. contributed to the discussion. All the authors approved the final version of the manuscript.

## Supporting information



Supporting Information

Supplemental Movie 1

Supplemental Movie 2

## Data Availability

The data that support the findings of this study are available from the corresponding author upon reasonable request.
