# Supplementary material for: Coordination‐Induced Magnetism Strategy for Highly Selective and Efficient Uranium Separation
Source: Adv Sci (Weinh). 2024 Nov 4;11(48):2408642. doi: 10.1002/advs.202408642 (PMC11672296; doi:10.1002/advs.202408642)
Supplement: Supplementary file 1 — Supporting Information [file ADVS-11-2408642-s002.docx]

Supporting Information

Coordination-Induced Magnetism Strategy for Highly Selective and Efficient Uranium Separation

*Shilei Zhao, Tiantian Feng, Jiacheng Zhang, Meng Cao, Lijuan Feng, Yue Ma, Tao Liu, Yihui Yuan* and Ning Wang**

Dr. S. Zhao, Dr. T. Feng, J. Zhang, Dr. M. Cao, Dr. L. Feng, Dr. Y. Ma, Prof. T. Liu, Prof. Y. Yuan* and Prof. N. Wang*

State Key Laboratory of Marine Resource Utilization in South China Sea, Hainan University, Haikou 570228, P. R. China

*E-mail: wangn02@foxmail.com (N. W.), yuanyh@hainanu.edu.cn (Y. Y.)


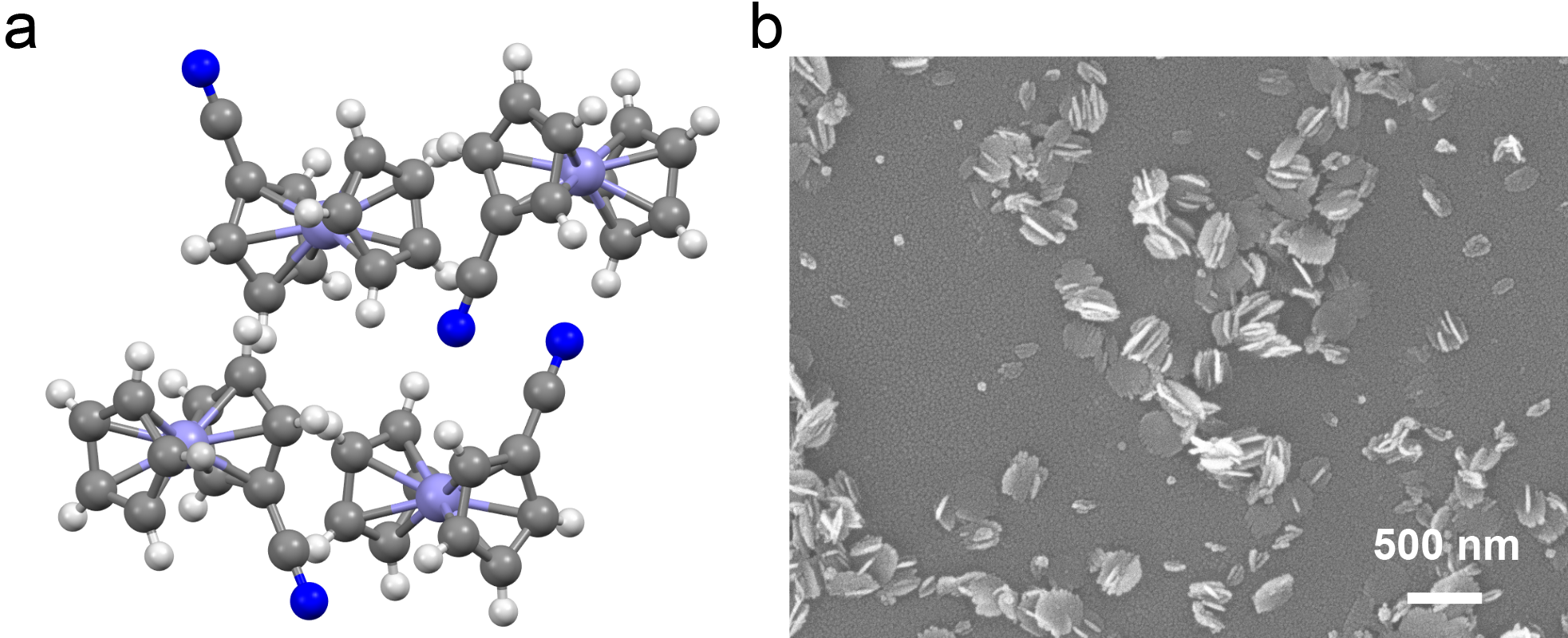


Figure S1. Crystal structure and SEM images of Fc-CN nanocrystal. a) Crystal structure of Fc-CN nanocrystal. b) SEM images of recrystalizated Fc-CN nanocrystals.


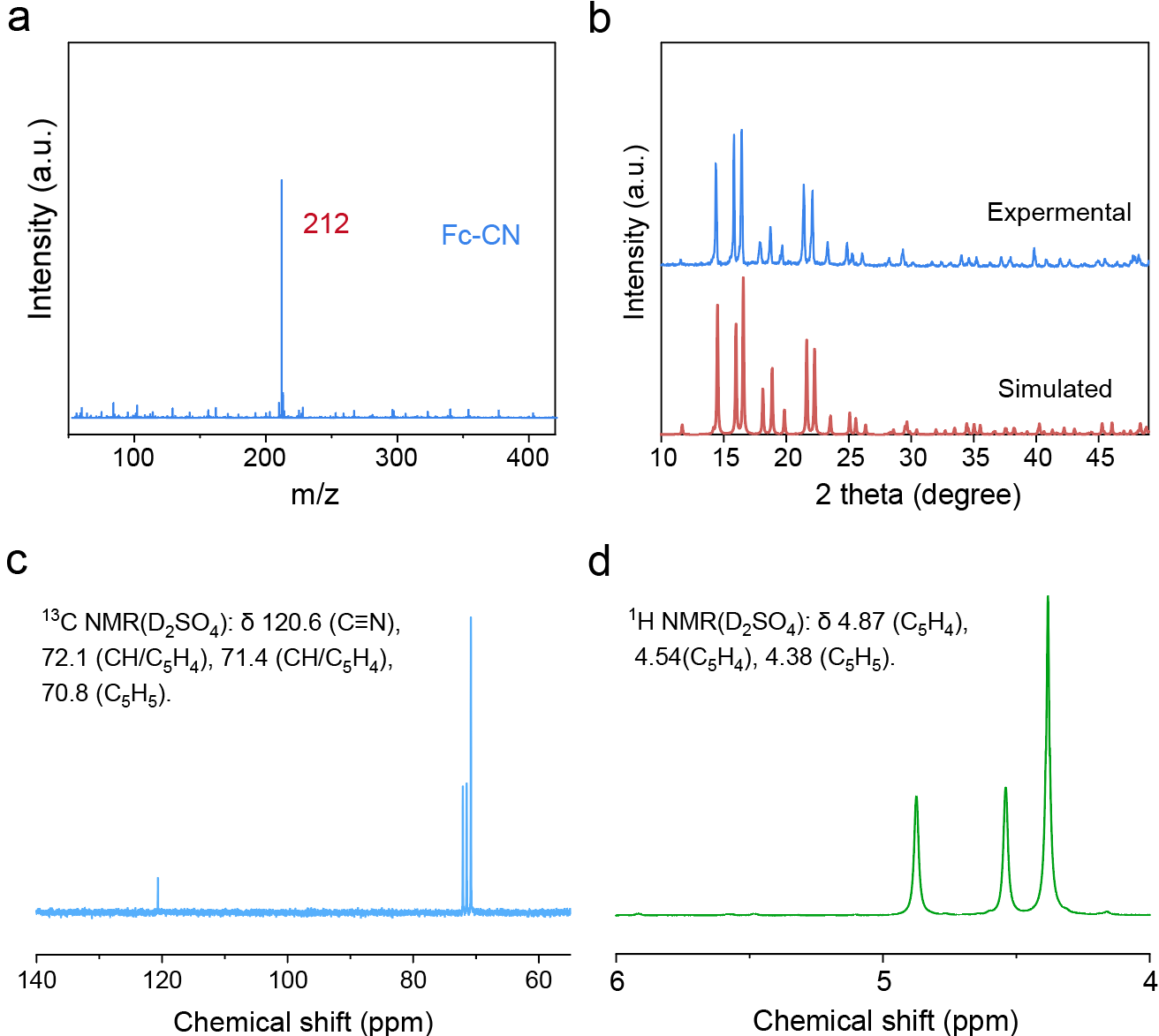


Figure S2. Characterization of Fc-CN. a) The mass spectrum of Fc-CN. b) Experimental (blue) and simulated (red) XRD patterns of Fc-CN nanocrystal. c) ^13^C NMR spectrum of Fc-CN. d) ^1^H NMR spectrum of Fc-CN.

The purification of Fc-CN is confirmed through the analysis of organic elemental analysis, mass spectrometry (MS), single-crystal X-ray diffraction, X-ray diffraction (XRD) patterns, and nuclear magnetic resonance (NMR) spectra. The chemical component analysis of the nanocrystal indicates the presence of C, H, N, and Fe with contents of 62.43%, 4.26%, 6.63%, and 26.5%, respectively, which match with the contents in the chemical formula C_11_H_9_NFe (Table S1, Supporting Information). MS analysis result reveals a prominent peak at 212, corresponding to the molecular weight of Fc-CN (Figure S2a, Supporting Information). The crystal structure can be verified by the excellent agreement between the experimental and simulated XRD patterns of Fc-CN nanocrystal (Figure S2b, Supporting Information). Single-crystal XRD analysis was employed to ascertain the structure of Fc-CN. The crystal structure of Fc-CN reveals a p212121 space group within the orthorhombic crystallized system (Table S2, Supporting Information). The ^13^C NMR spectrum of Fc-CN exhibits a distinct signal at 120.6 ppm, corresponding to the carbon atoms in the nitrile group of Fc-CN (Figure S2c, Supporting Information). And the peaks at 72.1, 71.4, and 70.8 ppm are attributable to the cyclopentadienyl carbon atoms. On the other hand, the ^1^H NMR spectrum of Fc-CN displays signals at 4.87, 4.54, and 4.38 ppm (Figure S2d, Supporting Information), which belong to hydrogen atoms from the cyclopentadienyl group. All these results prove the correct structure and the high purity of the Fc-CN nanocrystal.


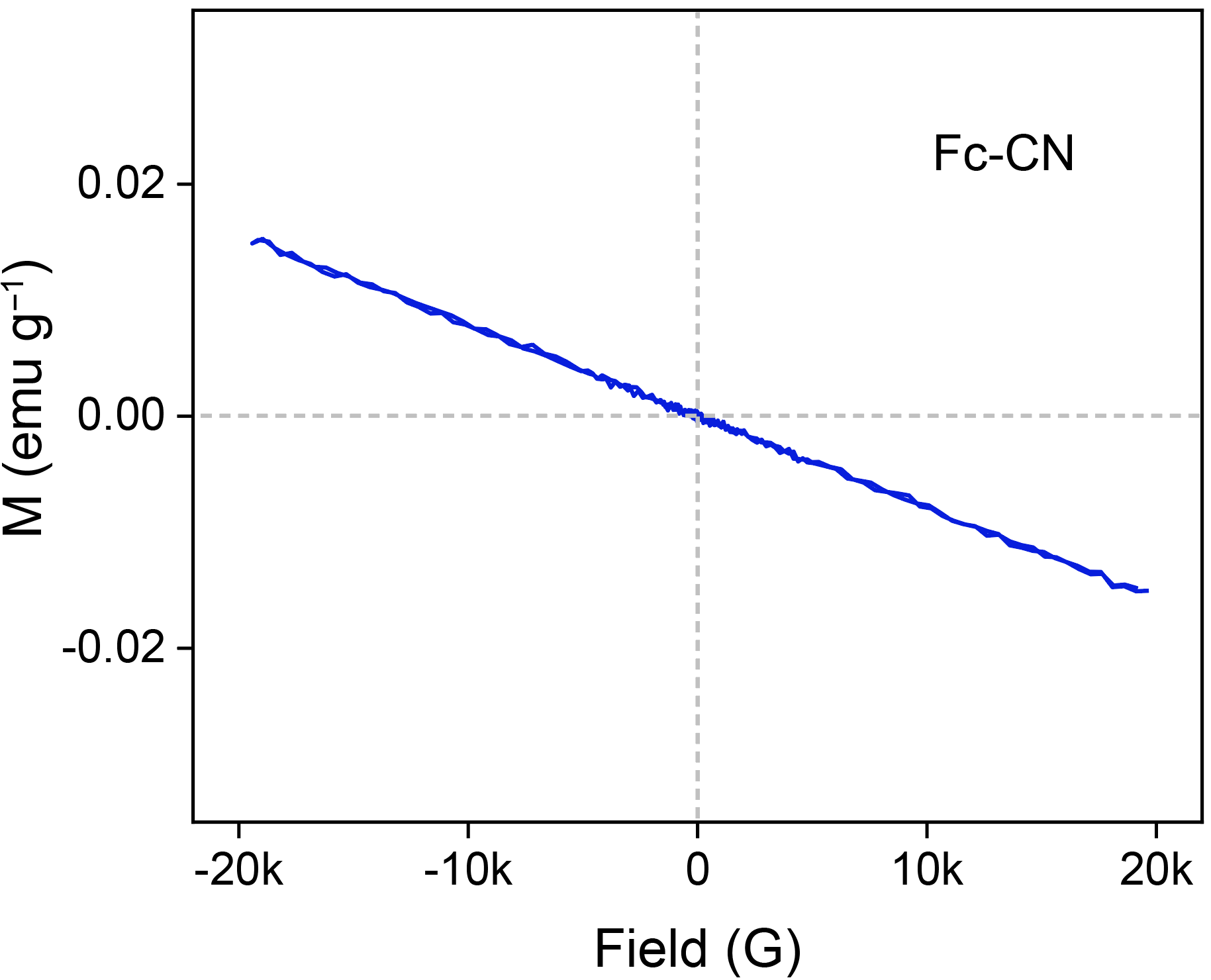


Figure S3. Magnetization curve of Fc-CN nanocrystals.


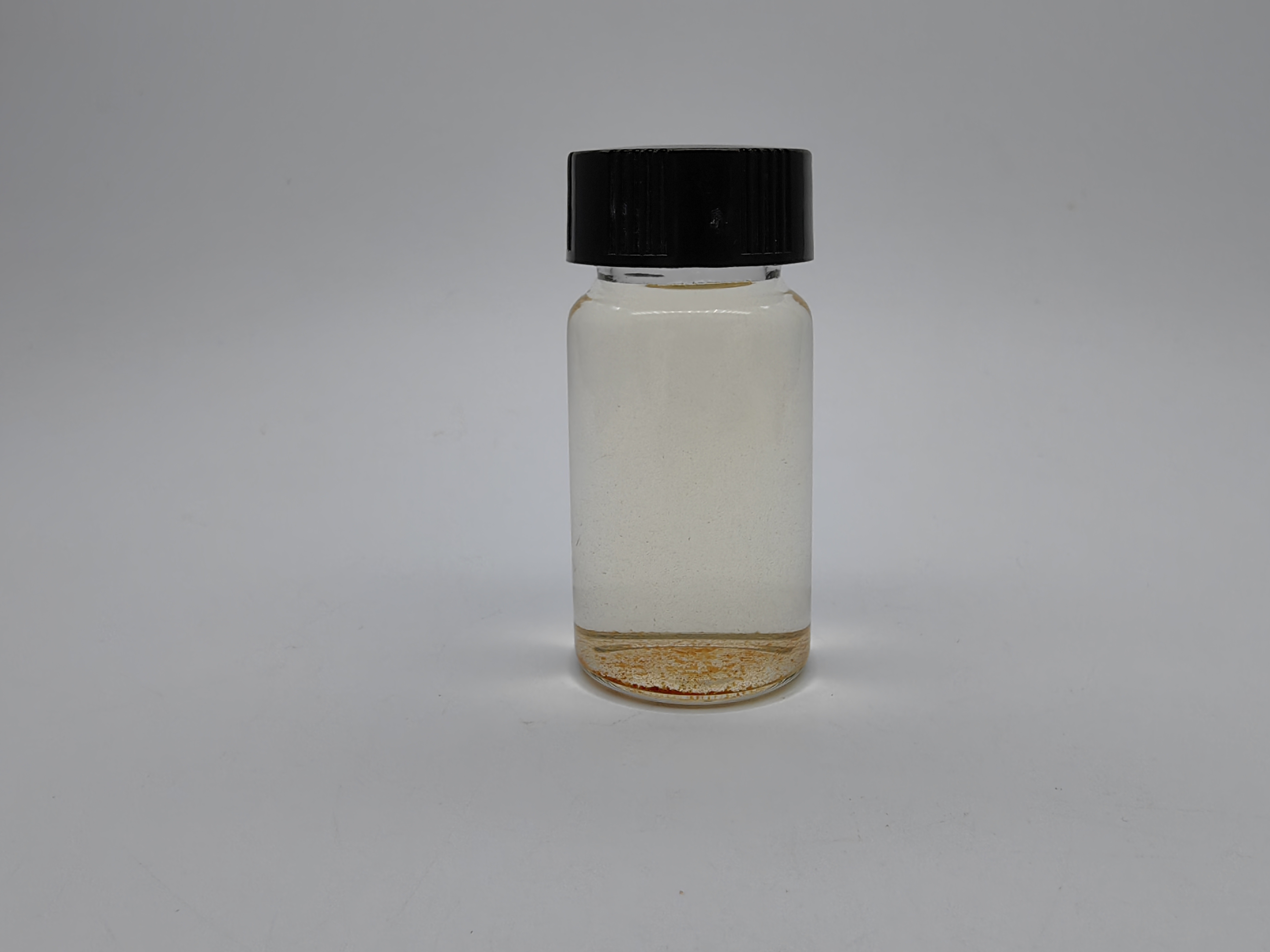


Figure S4. The photograph of Fc-CN nanocrystal without light irradiation.


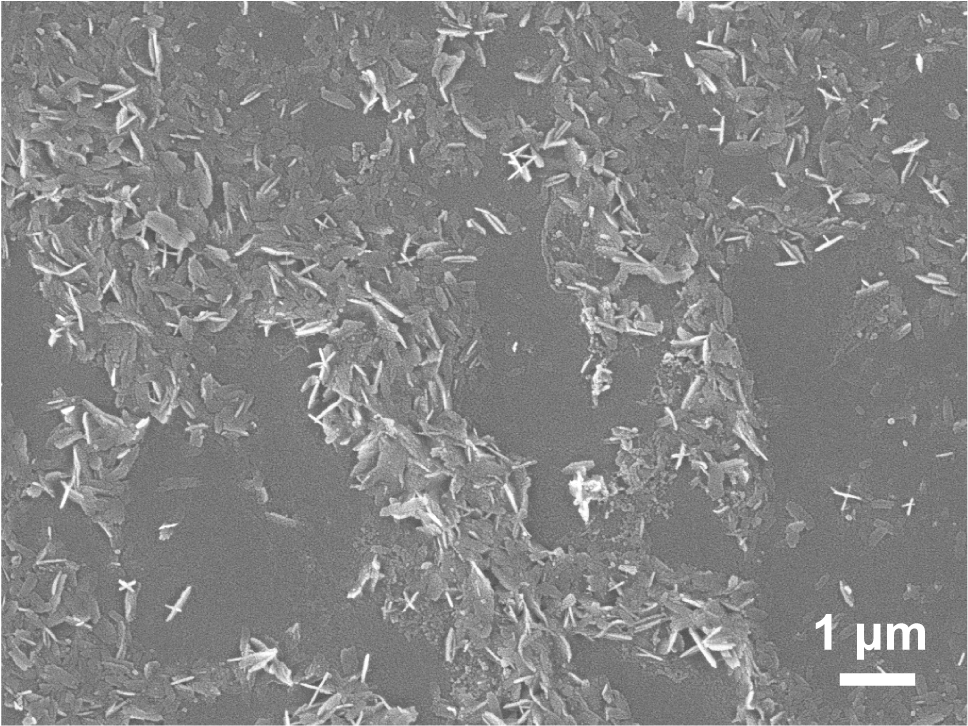


Figure S5. SEM image of Fc-CN-*L.*


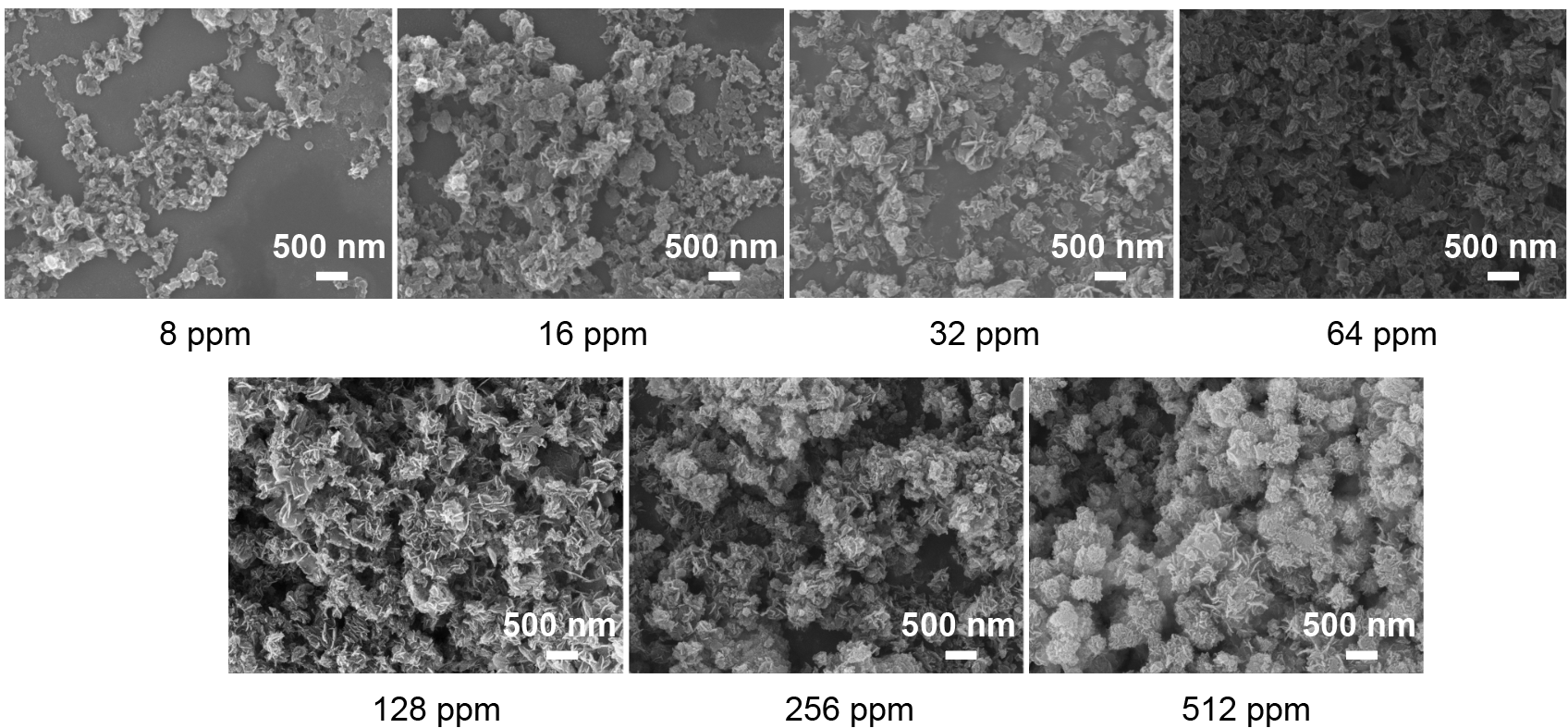


Figure S6. SEM images of U/Fc-CN-*L* in uranyl ion solution of different uranium concentrations.


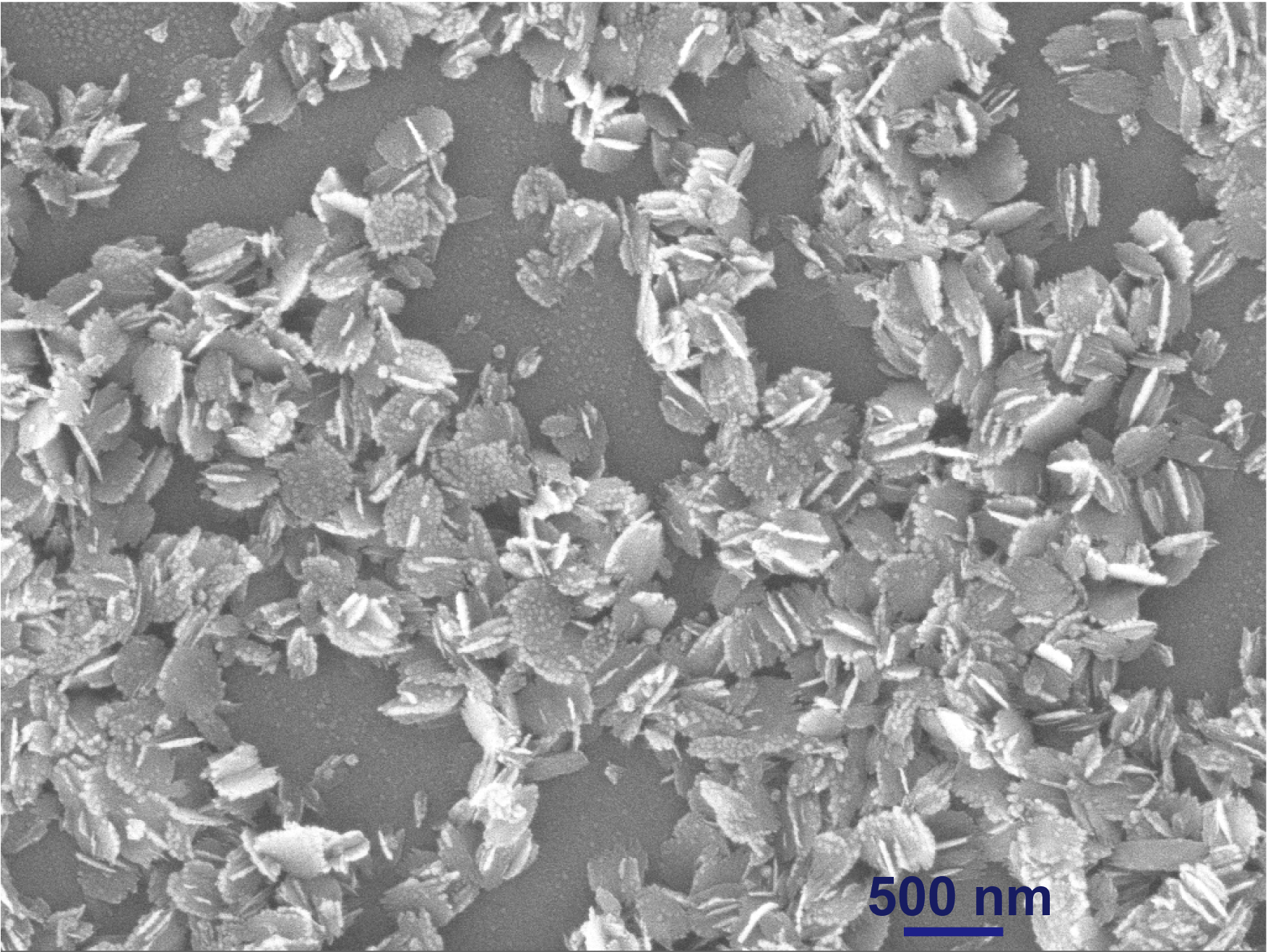


Figure S7. SEM image of U/Fc-CN-*D* in uranium solution without light irradiation.


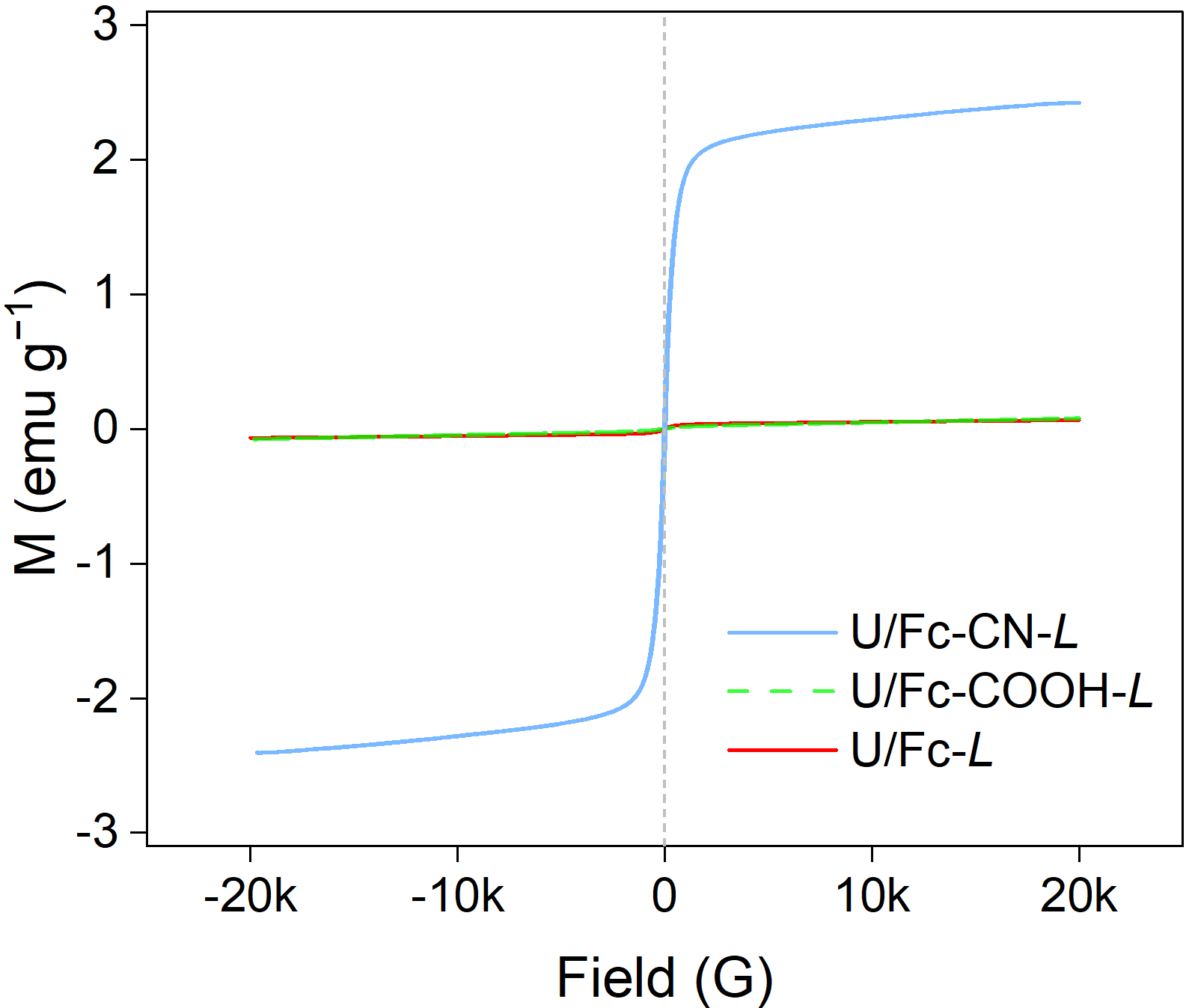


Figure S8. Magnetization curves of U/Fc-CN-*L*, U/Fc-COOH-*L*, and U/Fc-*L*.


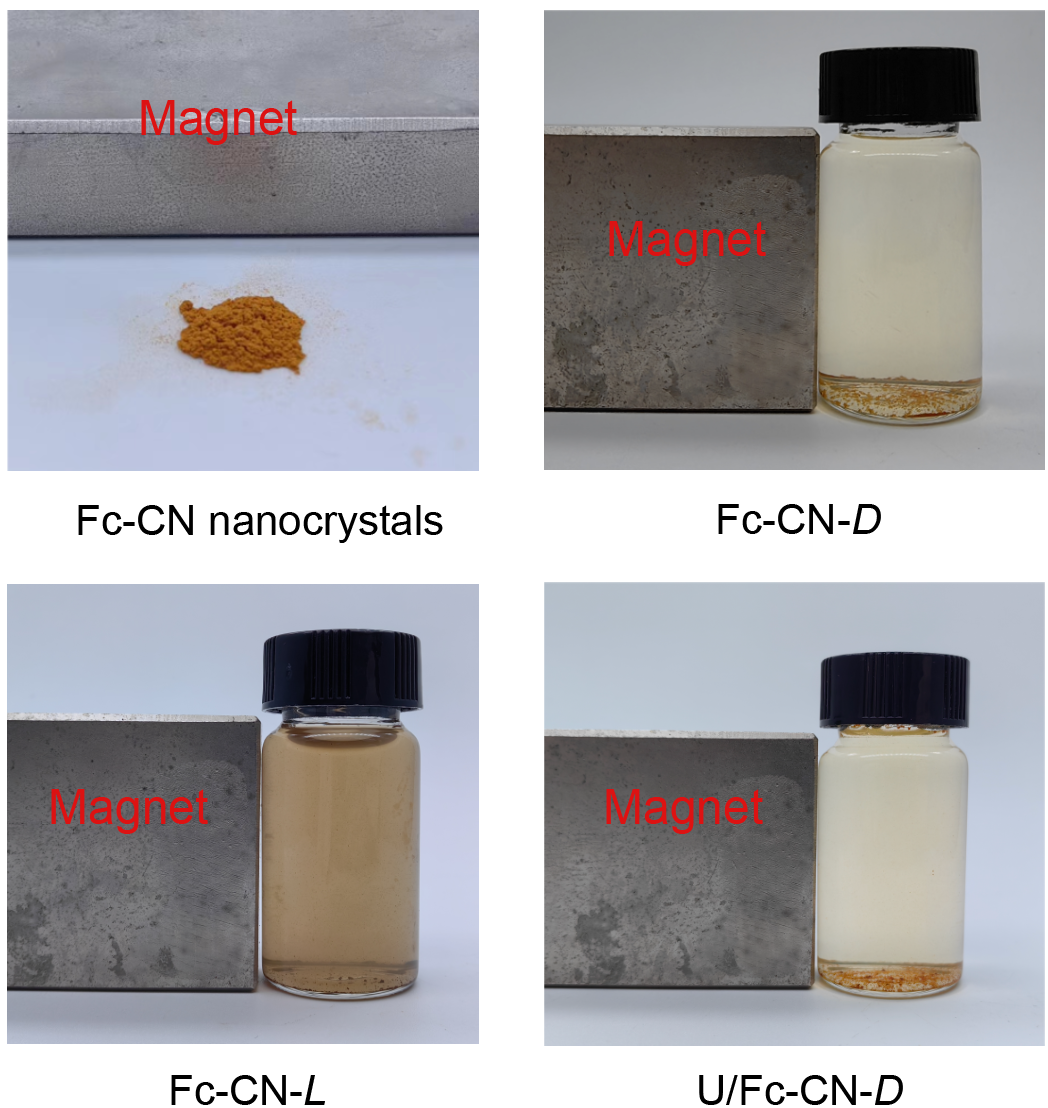


Figure S9. Magnetic response behaviors of Fc-CN nanocrystals, Fc-CN-*D*, Fc-CN-*L* and U/Fc-CN-*D.* These samples all lack magnetic separable ability. The magnet used in this analysis possesses a magnetic field strength of 0.3 T.


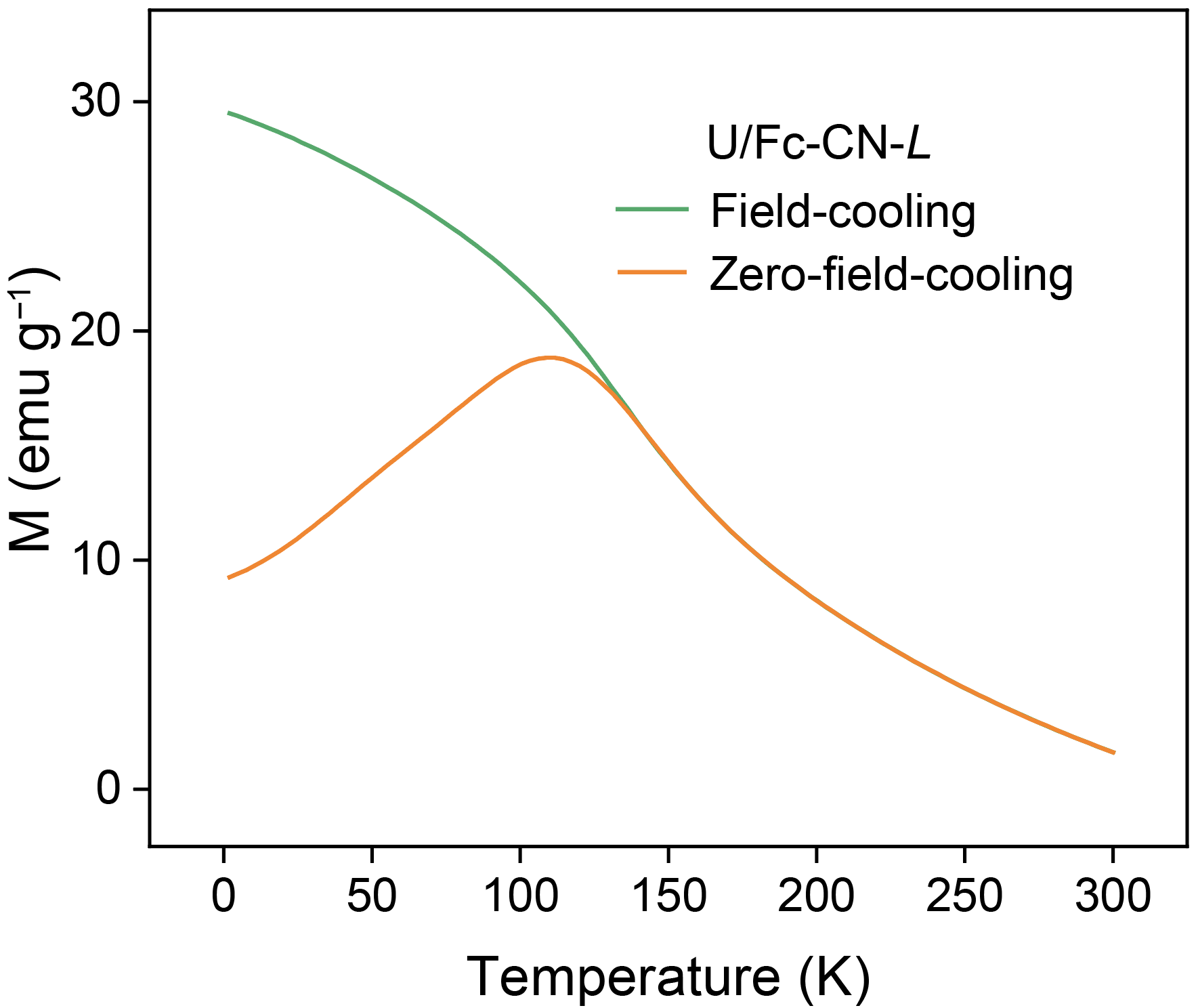


Figure S10. Field-cooling/zero-field-cooling magnetization curves of U/Fc-CN-*L*.


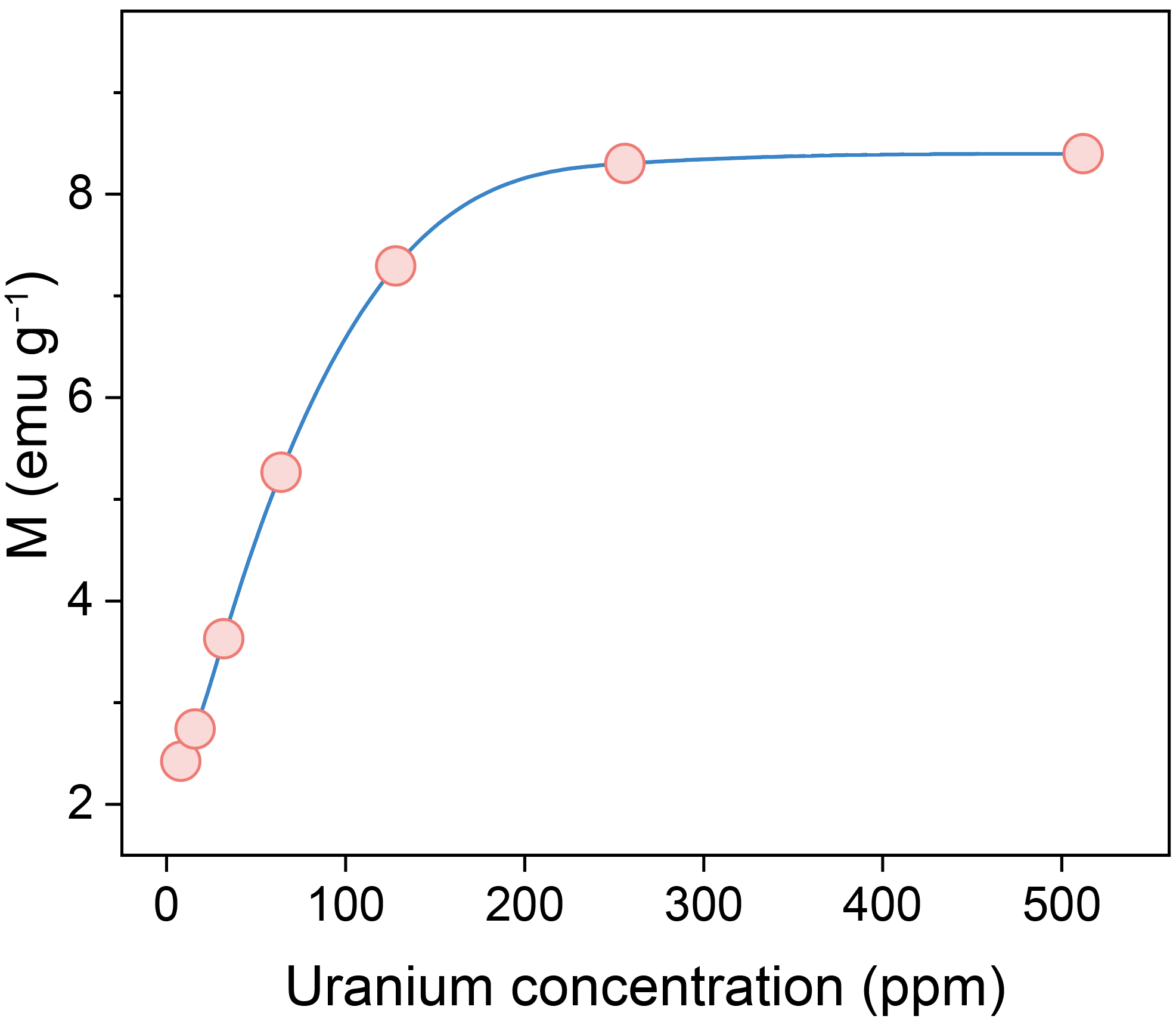


Figure S11. The influence of uranium concentration on the saturation magnetization.


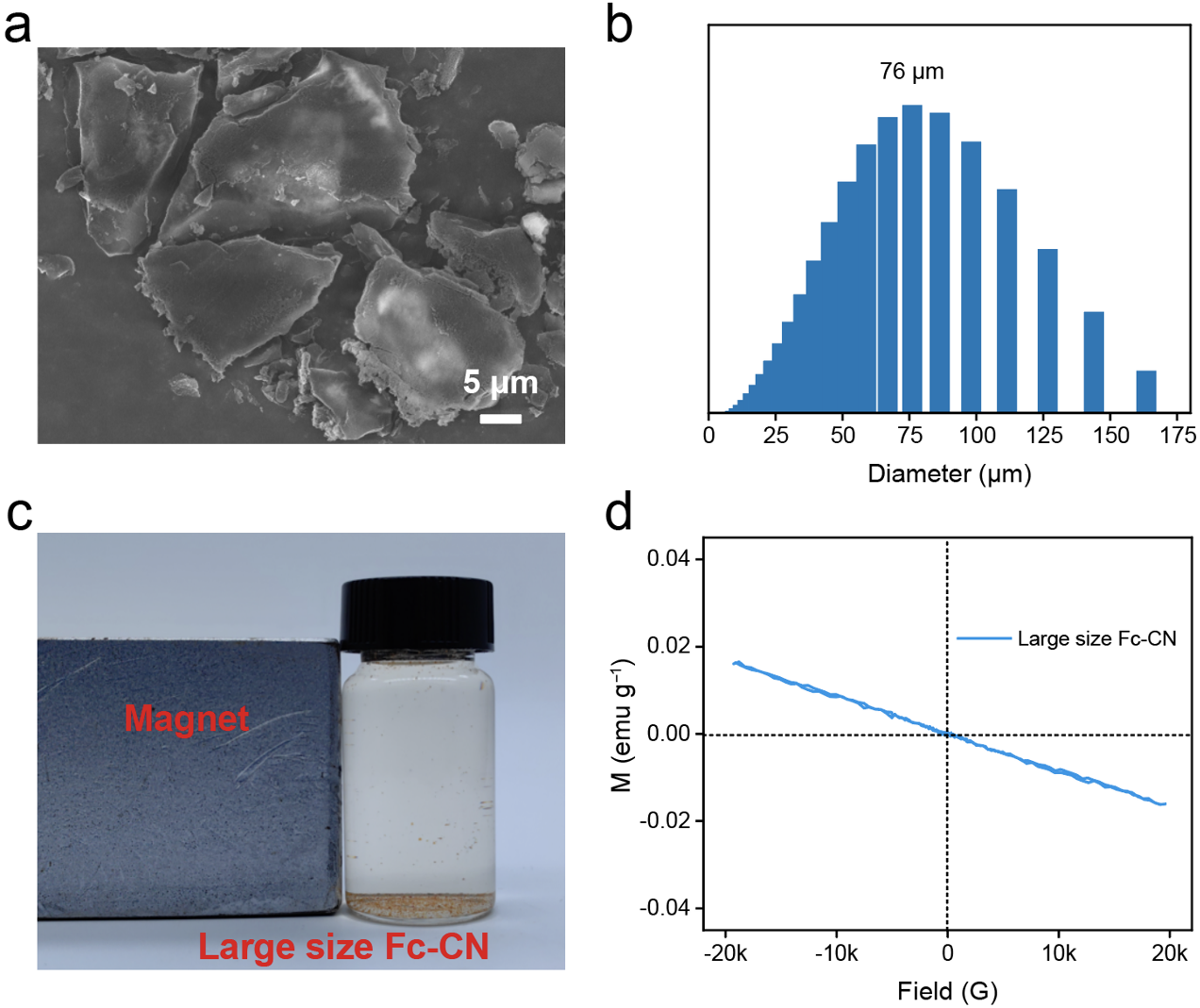


Figure S12. Particle size and corresponding magnetic characterization of Fc-CN crystal. a) SEM image of Fc-CN crystal with larger particle size. b) Particle size distribution Fc-CN crystal with larger particle size. c) Magnetic recovery behavior of Fc-CN crystals with larger particle size. d) Magnetization curves of Fc-CN crystals with larger particle size.


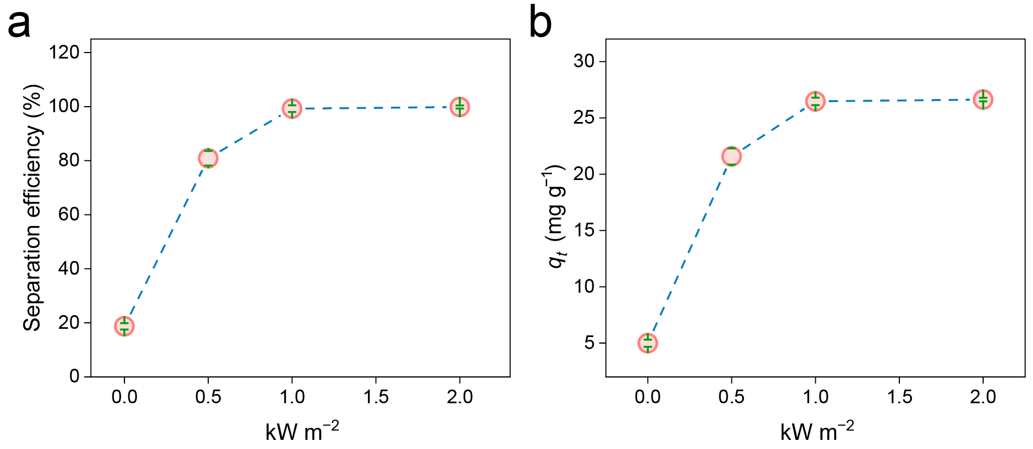


Figure S13. Uranium separation efficiency (a) and adsorption capacity (b) of Fc-CN nanocrystal under different light intensities in 8 ppm uranium solution at pH 5. The data are presented as mean ± standard deviation (SD).


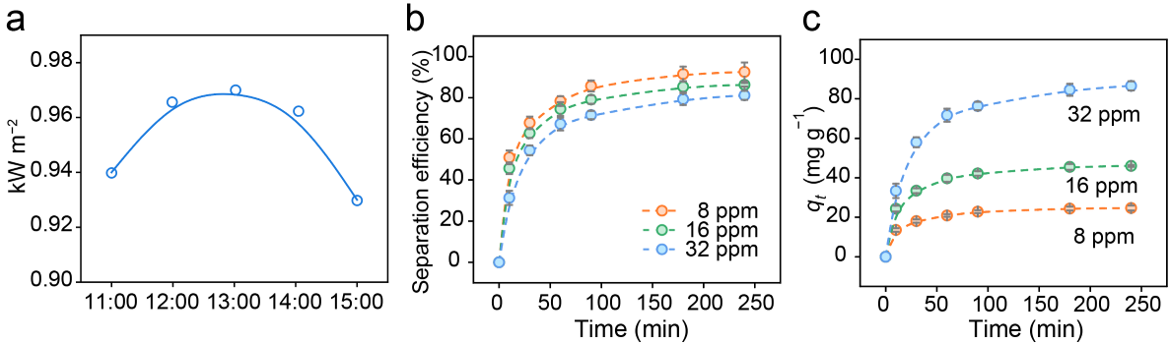


Figure S14. Separation of uranium by Fc-CN nanocrystals under natural sunlight irradiation. a) The change of light intensity during the test under natural sunlight irradiation. Uranium separation kinetics (b) and adsorption capacity (c) of Fc-CN nanocrystals under natural sunlight irradiation. The pH of the used uranium solution is pH 5, and 6 mg Fc-CN nanocrystals was added to 20 mL uranyl nitrate solutions of different uranium concentrations, respectively. The data are presented as mean ± standard deviation (SD).


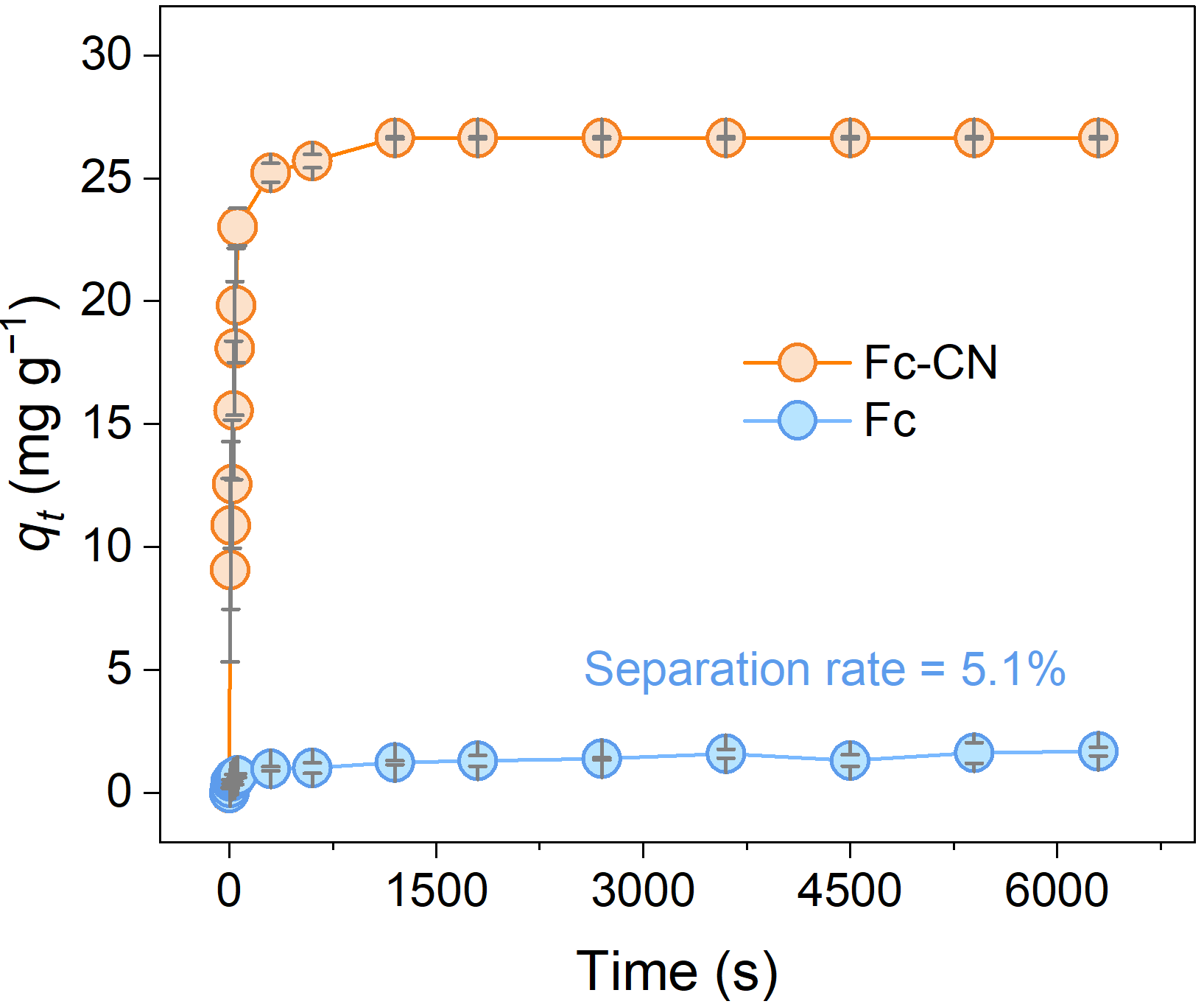


Figure S15. Kinetics of uranium separation by Fc-CN nanocrystals and Fc from uranyl solution (8 ppm) under 1 kW m^−2^ light irradiation. The data are presented as mean ± standard deviation (SD).


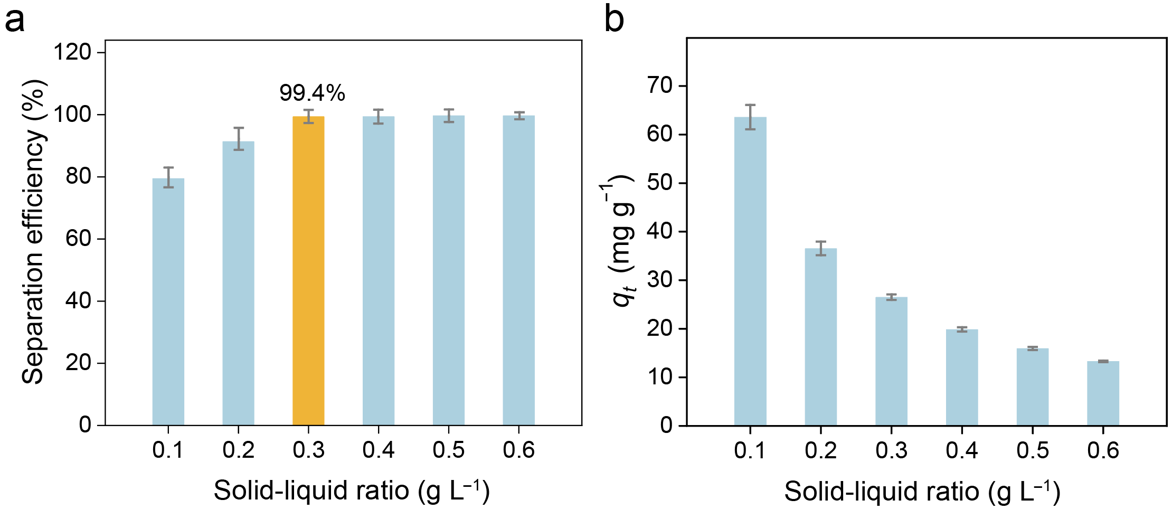


Figure S16. The uranium separation efficiency (a) and adsorption capacity (b) of Fc-CN nanocrystals with different dosage in 8 ppm uranium solution at pH 5. The data are presented as mean ± standard deviation (SD).


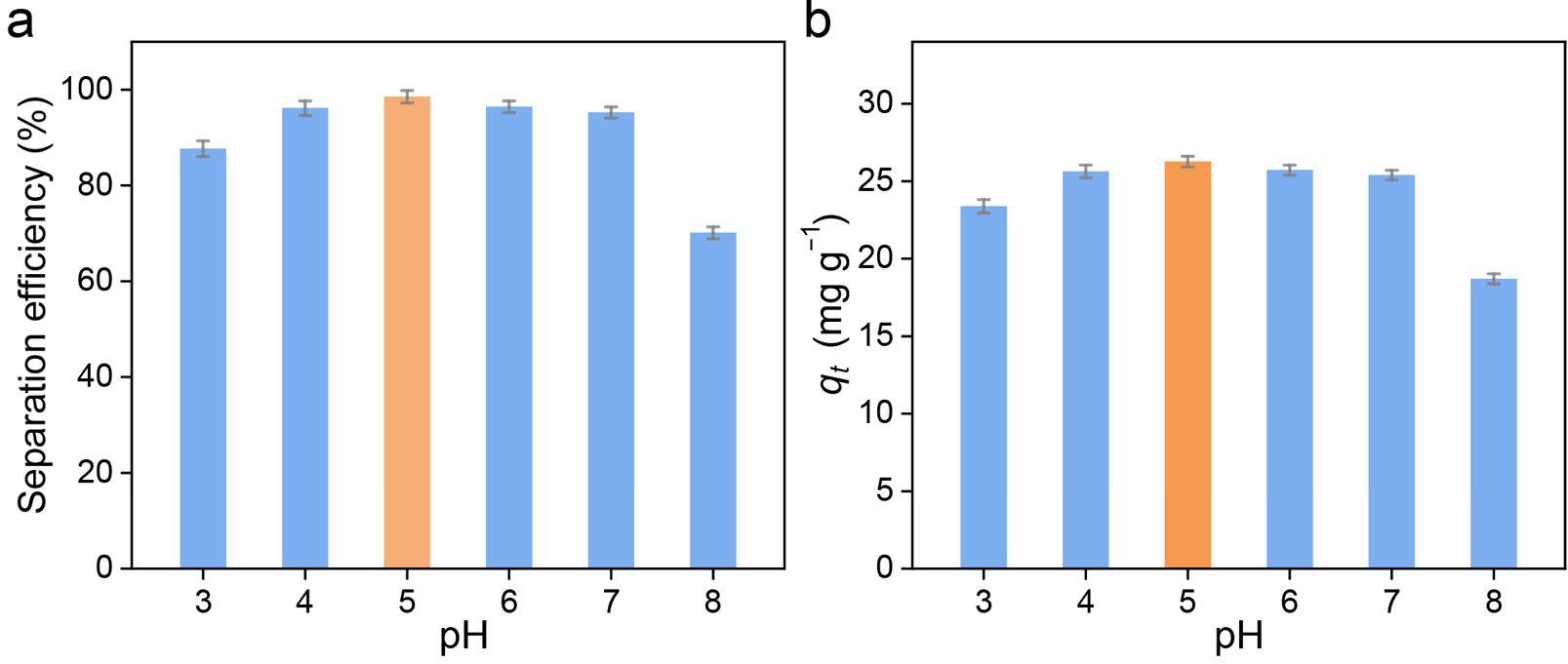


Figure S17. The uranium separation efficiency (a) and adsorption capacity (b) of Fc-CN nanocrystals with different pH value in 8 ppm uranium solution, respectively. The data are presented as mean ± standard deviation (SD). The results indicate that as the pH increases from 3 to 5, the uranium separation efficiency improves significantly, reaching an optimal removal rate of nearly 100% at pH 5. However, when the pH is further increased to 8, a noticeable decline in separation efficiency is observed, with the removal rate dropping to approximately 70%. This pH dependent uranium separation performance may be attributed to the altered speciation of uranium ions.


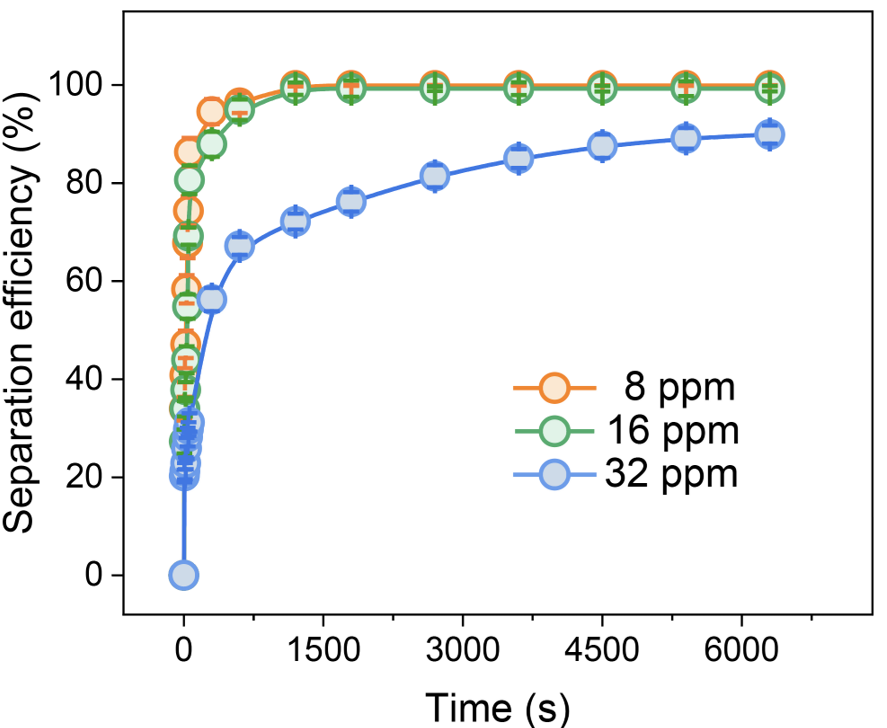


Figure S18. Separation kinetics of uranium from a uranyl solution by Fc-CN nanocrystals (0.3 g L^−1^) at pH 5.0 under 1 kW m^−2^ light irradiation at 298.15 K. The data are presented as mean ± standard deviation (SD).


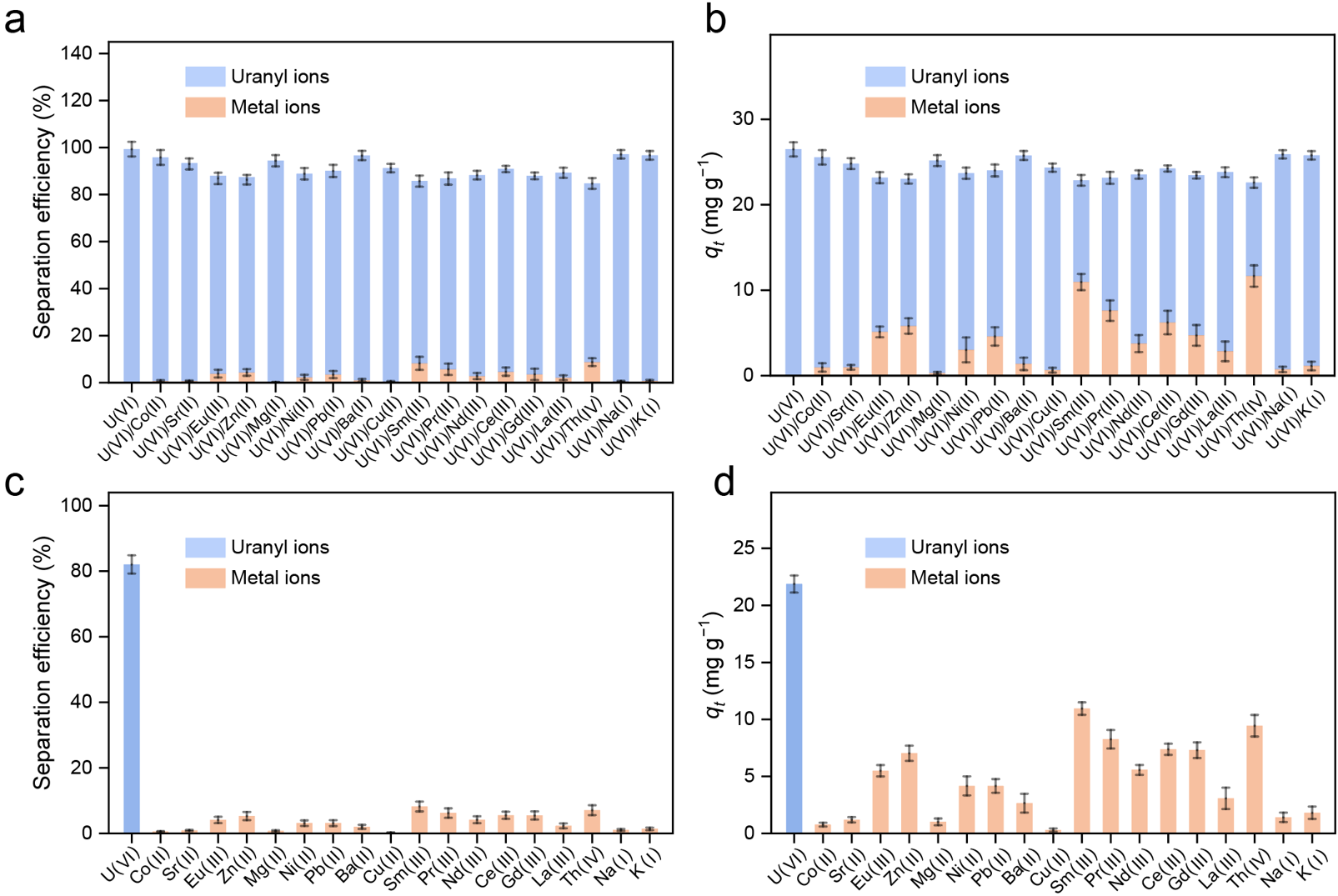


Figure S19. Separation selectivity of Fc-CN nanocrystals. a) The uranium separation efficiency and (b) adsorption capacity of Fc-CN nanocrystals in the presence of individual competitive metal ions at four times higher concentrations. c) Separation efficiency and (d) adsorption capacity of Fc-CN nanocrystals to uranium from solution containing multiple coexisting metal ions. The concentration of uranium is 8 ppm and the concentrations of the other metal ions are 40 ppm. The data are presented as mean ± standard deviation (SD).


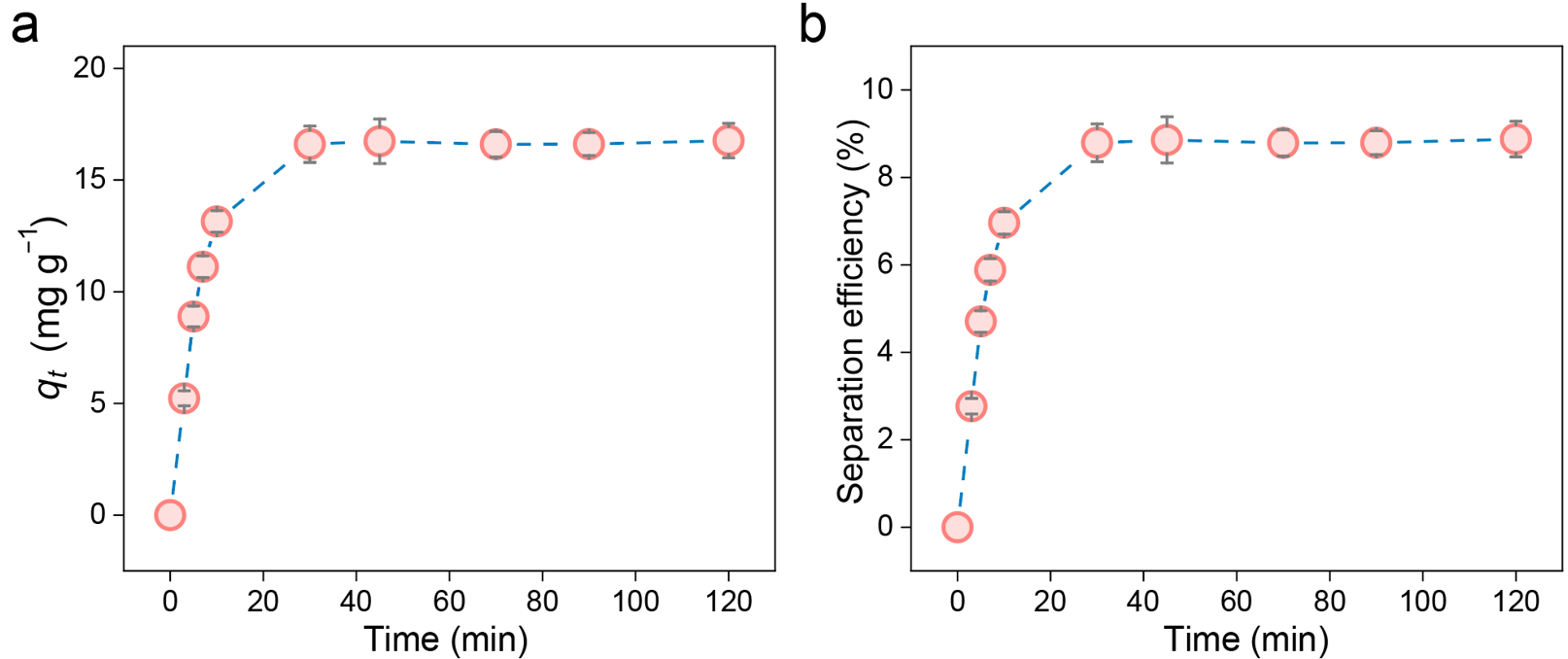


Figure S20. Uranium adsorption capacity (a) and separation kinetics (b) by Fc-CN nanocrystals from simulated nuclear wastewater with an initial uranium concentration of 18.9 ppm and pH 5.0 at 298.15 K under dark condition. The data are presented as mean ± standard deviation (SD).


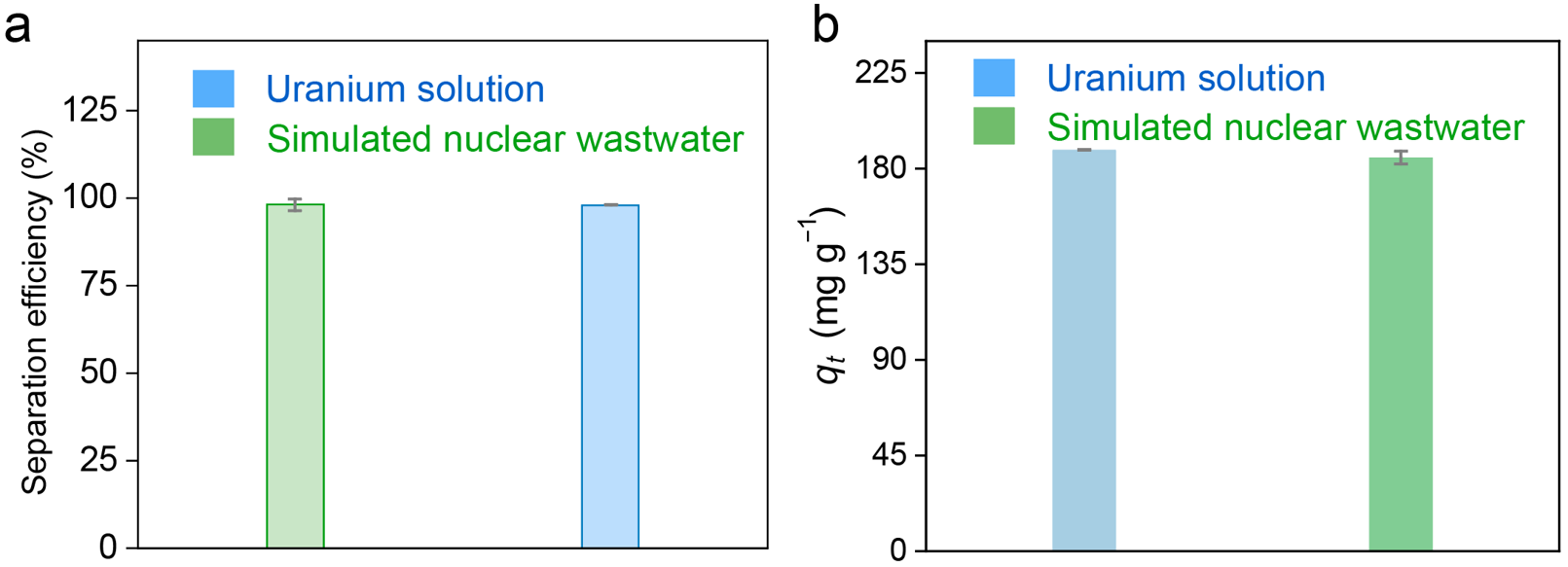


Figure S21. Uranium separation efficiency (a) and adsorption capacity (b) of Fc-CN nanocrystal in simulated nuclear wastewater and in uranium solution with the same uranium concentration of 18.9 ppm. The data are presented as mean ± standard deviation (SD).


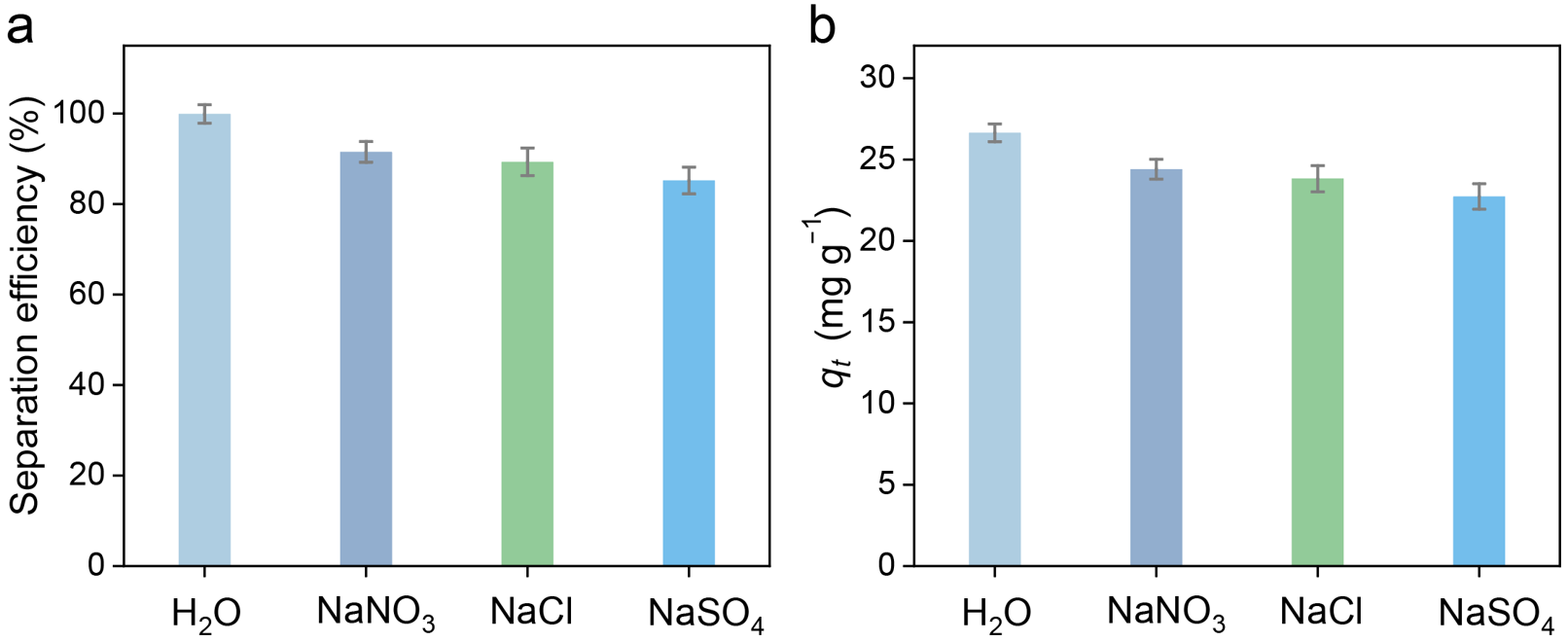


Figure S22. Influence of anions on the uranium separation efficiency (a) and adsorption capacity (b) of Fc-CN nanocrystals in uranium solution containing different anions of 10 mmol/L NaNO_3_, 10 mmol/L NaCl, and 5 mmol/L Na_2_SO_4_. The data are presented as mean ± standard deviation (SD).


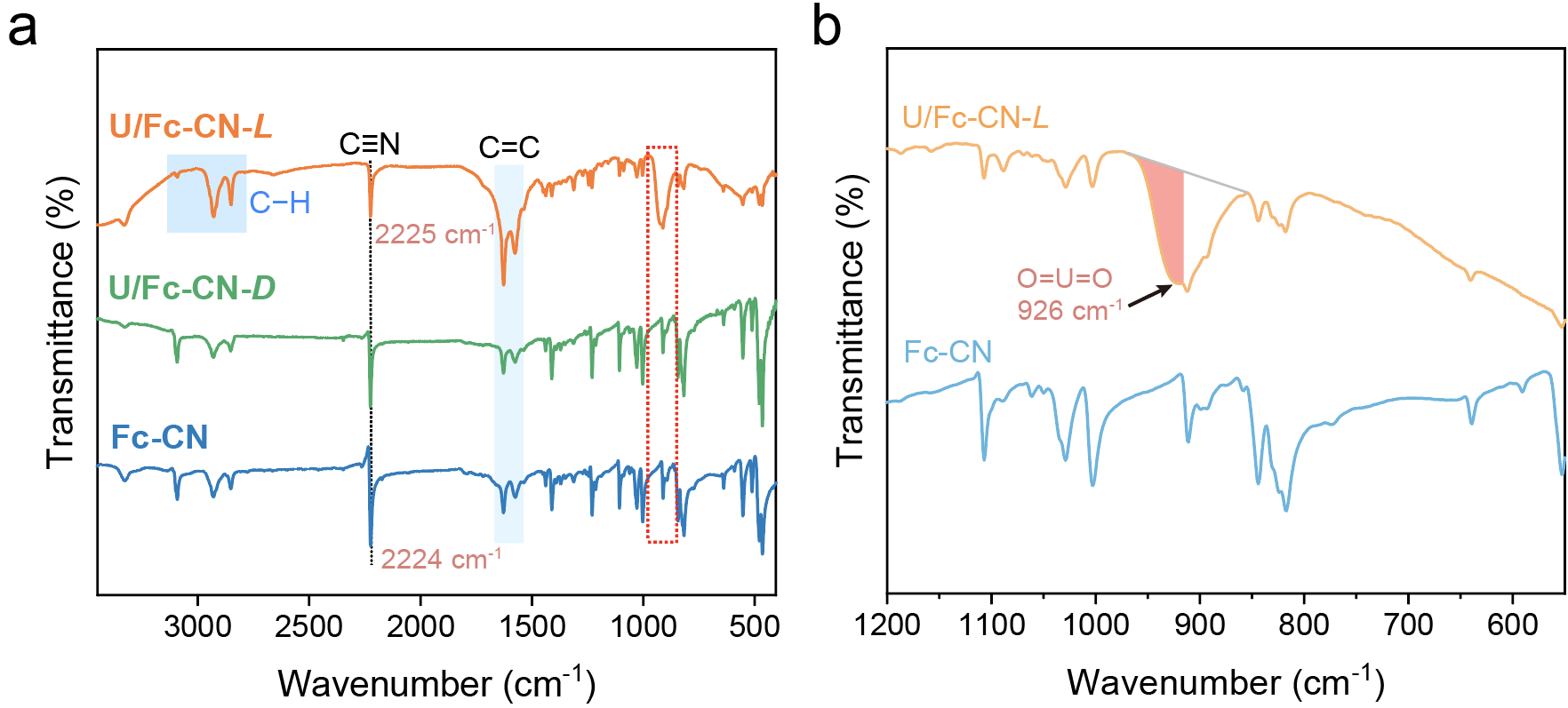


Figure S23. a) FTIR spectra of Fc-CN nanocrystals, U/Fc-CN-*D*, and U/Fc-CN-*L*. b) FTIR spectrum of U/Fc-CN-*L* in the wavelength range from 1200 to 550 cm^−1^.


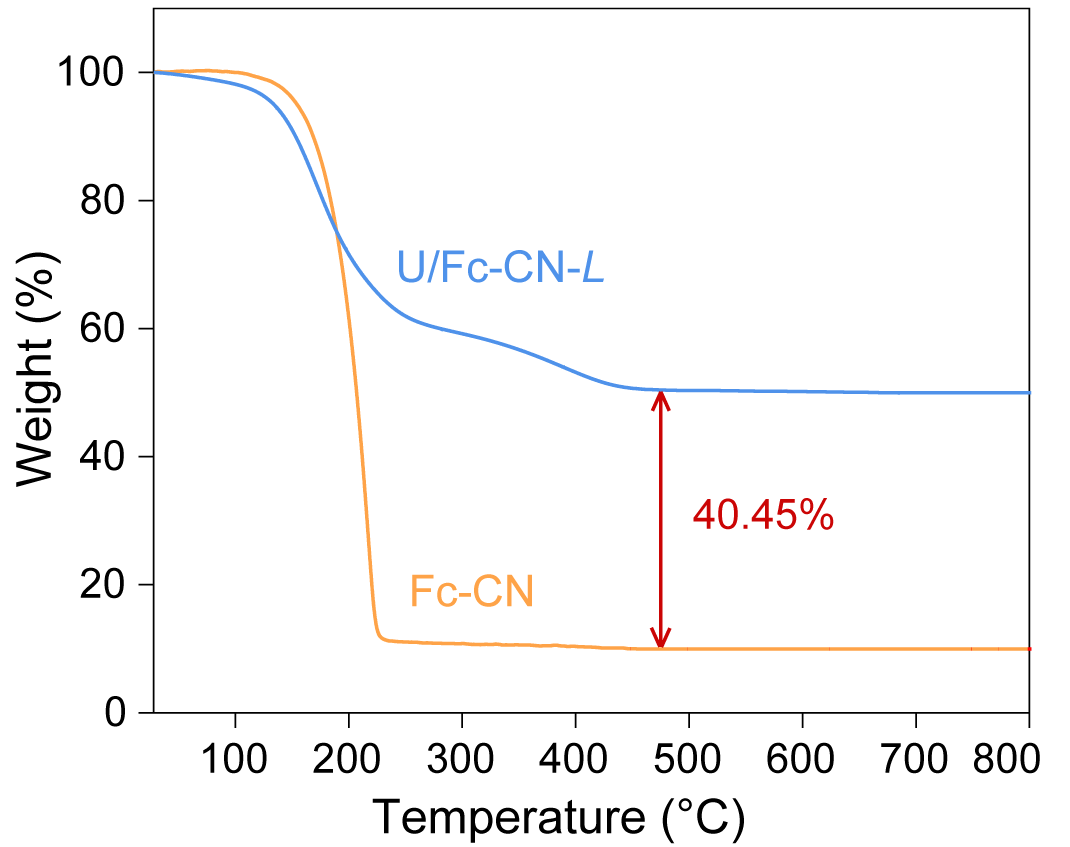


Figure S24. TGA spectra of Fc-CN and U/Fc-CN-*L*.


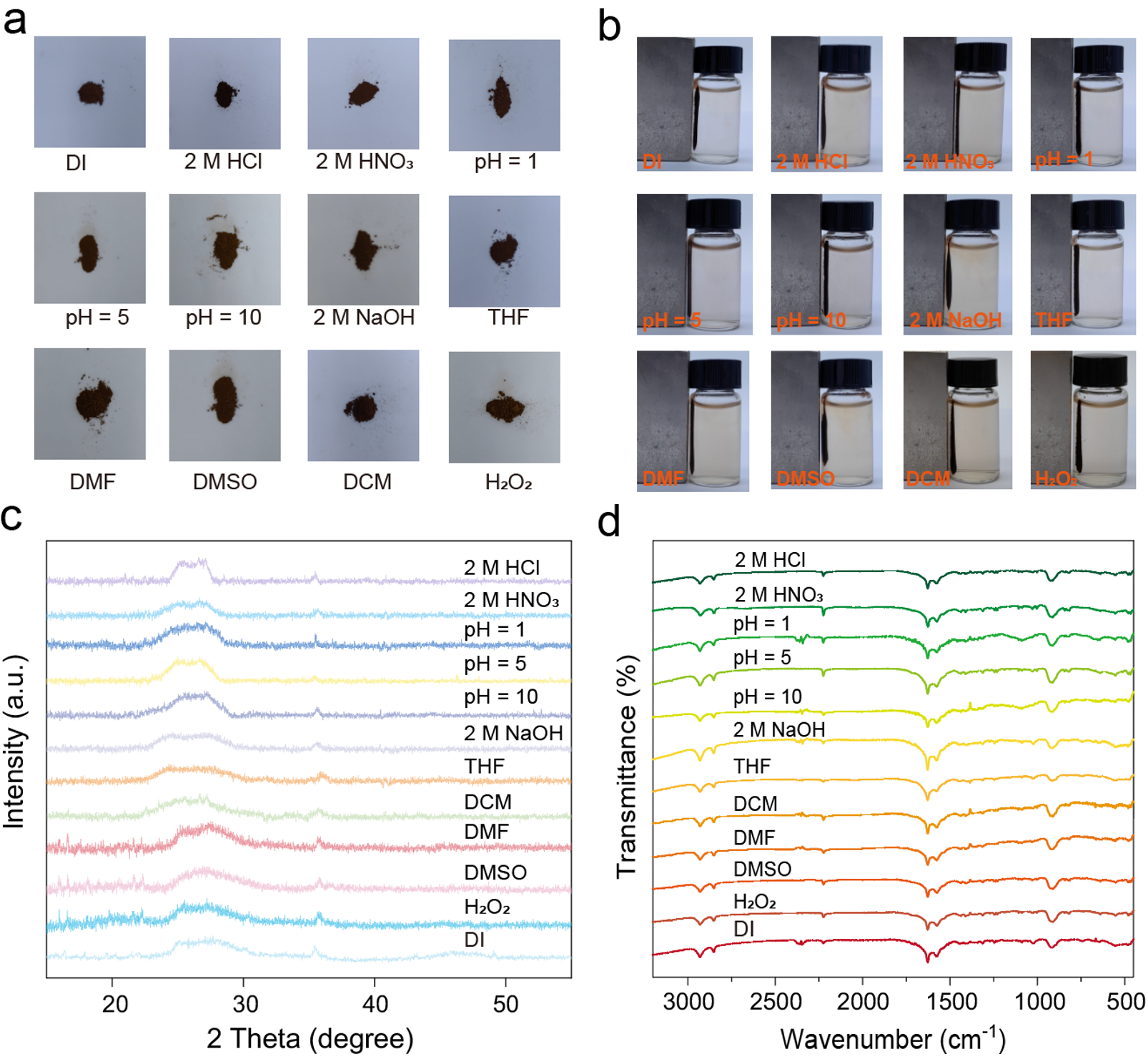


Figure S25. Chemical stability of U/Fc-CN-*L* aggregates with different treatments for five days. a) Morphology of U/Fc-CN-*L* powders with different treatments for five days. b) Magnetic performances of U/Fc-CN-*L* after different treatments for five days. XRD (c) and FTIR (d) spectra of U/Fc-CN-*L* with different treatments for five days. HCl: hydrochloric acid, HNO_3_: nitric acid, NaOH: sodium hydroxide, THF: tetrahydrofuran, DCM: dichloromethane, DMF: N, N-dimethylformamide, DMSO: dimethyl sulfoxide, H_2_O_2_: hydrogen peroxide, DI: deionized water. These results all proves the high stability of the U/Fc-CN-*L* aggregates.


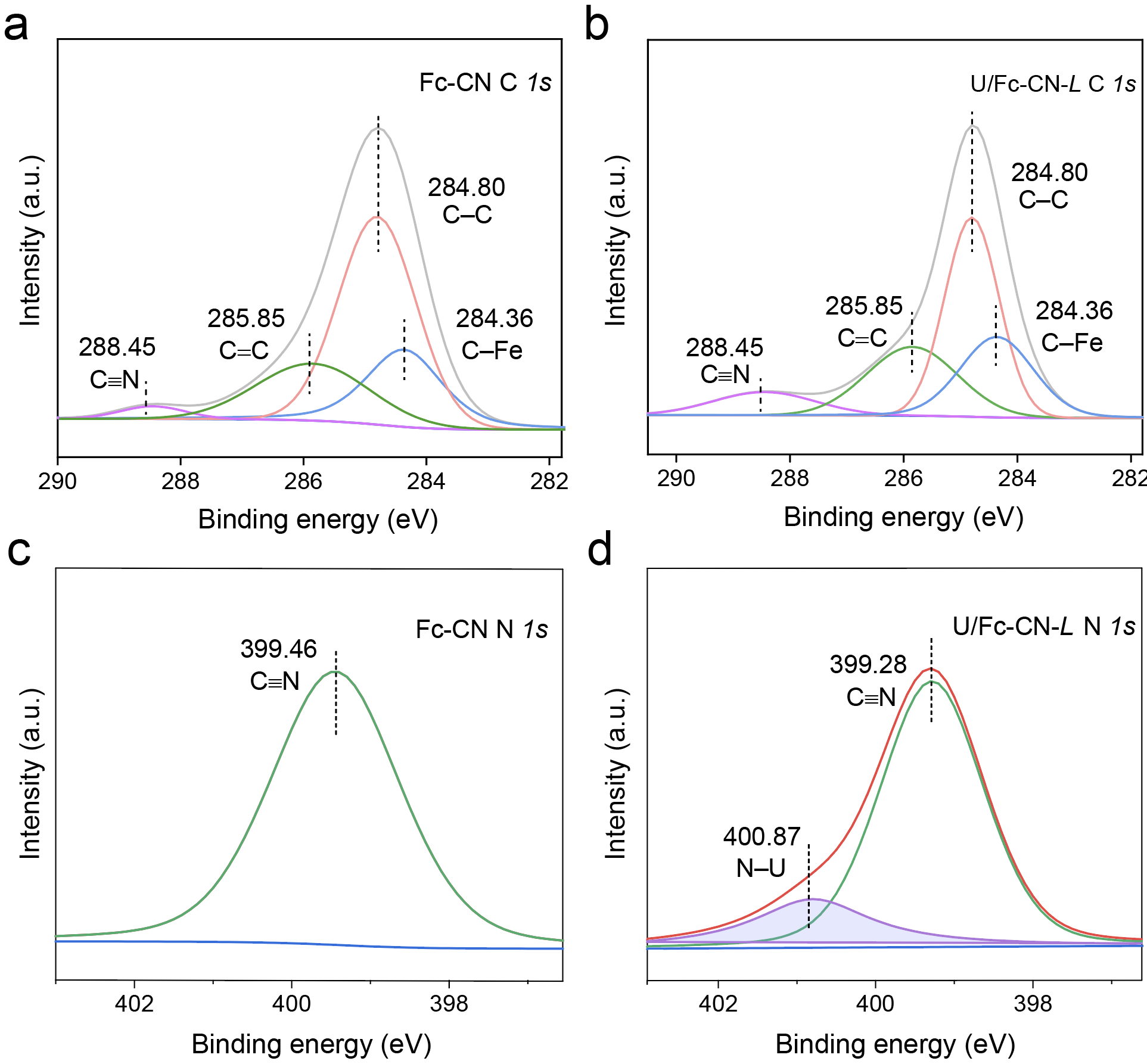


Figure S26. The high-resolution C *1s* spectra of Fc-CN a) and U/Fc-CN-*L* b), and N *1s* XPS spectra of Fc-CN c) and U/Fc-CN-*L* d), respectively. The high-resolution C *1s* spectrum exhibits four peaks corresponding to C–C, C≡N, C–Fe, and C=C, and the binding energies of the C–C bonds in both Fc-CN and U/Fc-CN-*L* are consistent at 284.80 eV. The high-resolution N *1s* spectrum of Fc-CN exhibits a peak at 399.46 eV, which corresponds to the C≡N group. While the high-resolution N *1s* spectra of U/Fc-CN-*L*, in addition to the C≡N (399.28 eV) peak, also displays a peak at 400.87 eV, indicating the presence of N–U bonds. These results suggest that the binding of uranium in U/Fc-CN-*L* is through the formation of N–U bonds.


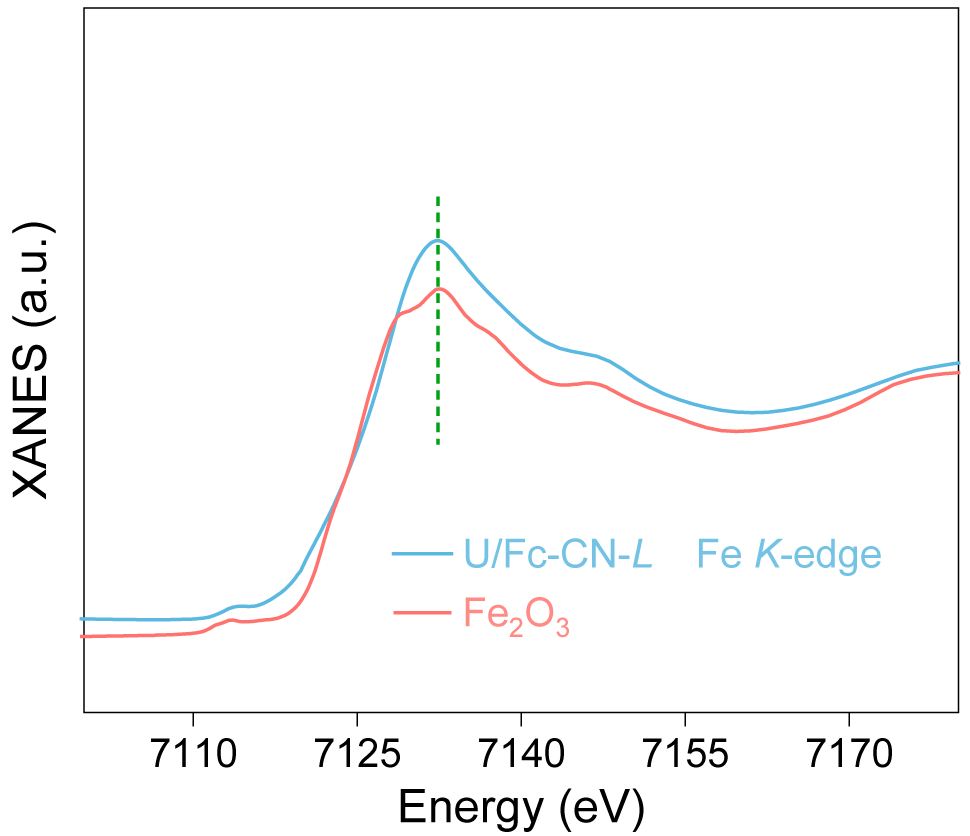


Figure S27. Fe *K*-edge XANES spectra of U/Fc-CN-*L* and Fe_2_O_3_.


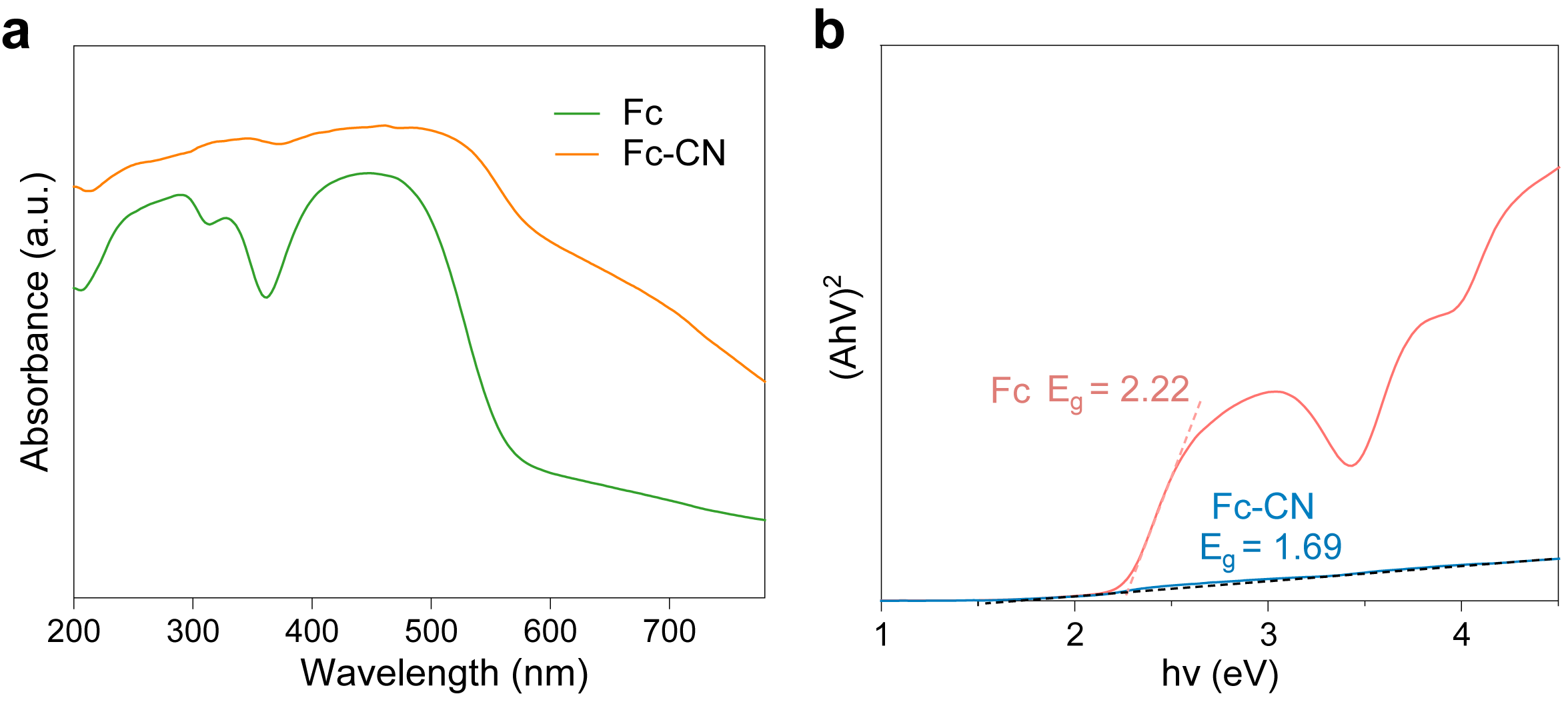


Figure S28. Optical character of Fc-CN and Fc. a) UV-vis DRS spectra of Fc and Fc-CN. b) The bandgaps of Fc and Fc-CN calculated by Tauc-Plot analysis. The ultraviolet-visible diffuse reflectance spectroscopy (UV-vis DRS) analysis reveals that, compared with Fc, Fc-CN exhibits a higher light absorption ability across a wide spectral range, and the corresponding bandgap width decreases from 2.22 eV for Fc to 1.69 eV for Fc-CN, indicating that the C≡N group in Fc-CN can facilitate the excitation of electrons.


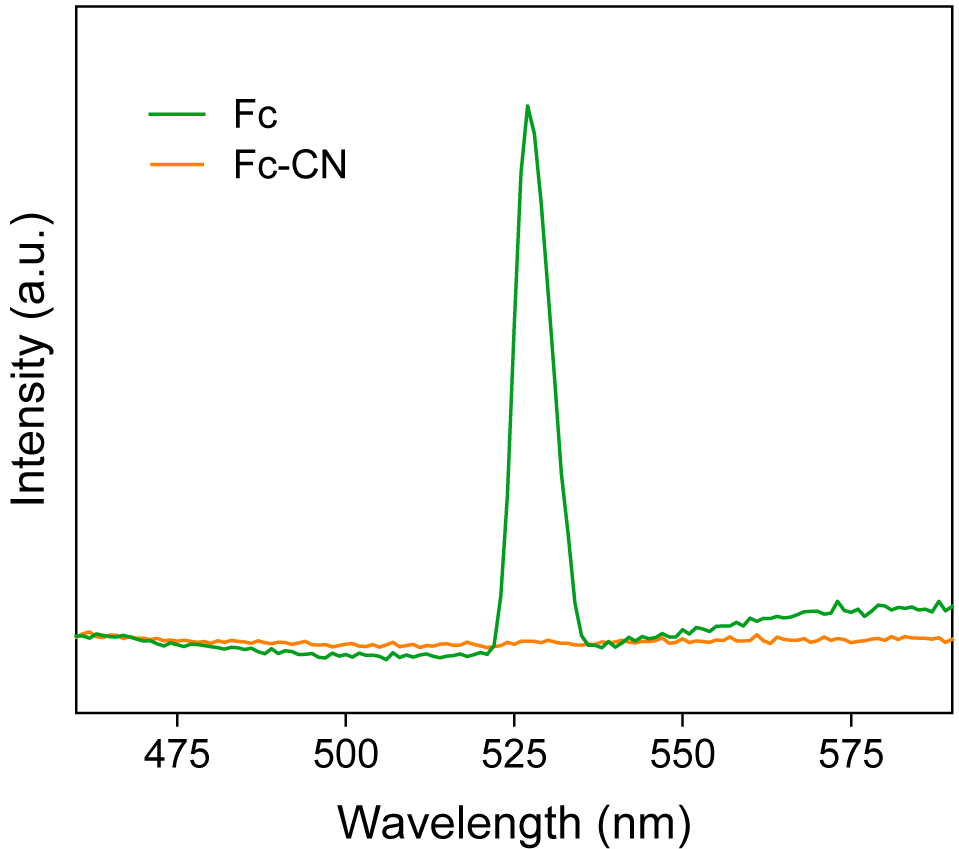


Figure S29. PL spectra of Fc and Fc-CN. The photoluminescence (PL) test shows that Fc-CN exhibits a significantly lower fluorescence intensity than Fc, suggesting that the electron-hole pair recombination rate in Fc-CN is lower.


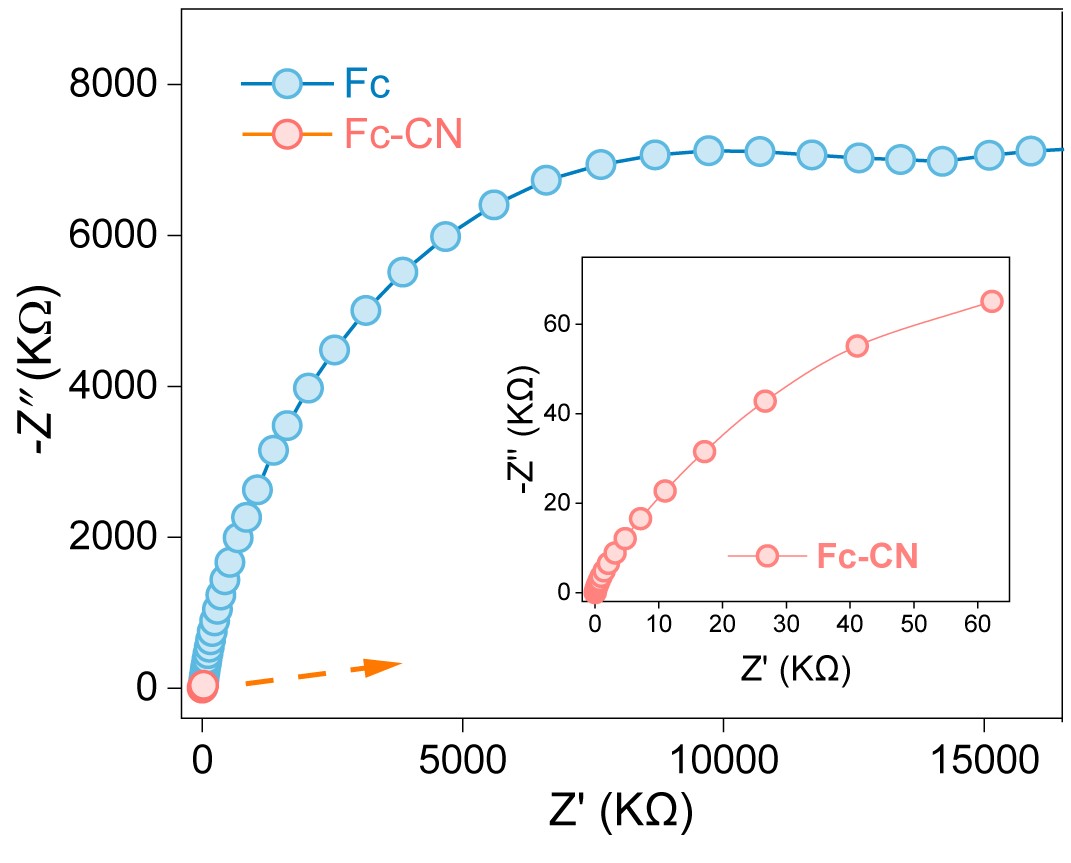


Figure S30. EIS of Fc and Fc-CN. The inset is an enlarged view of Fc-CN. EIS analysis further proves the higher charge separation and migration ability of Fc-CN than Fc.


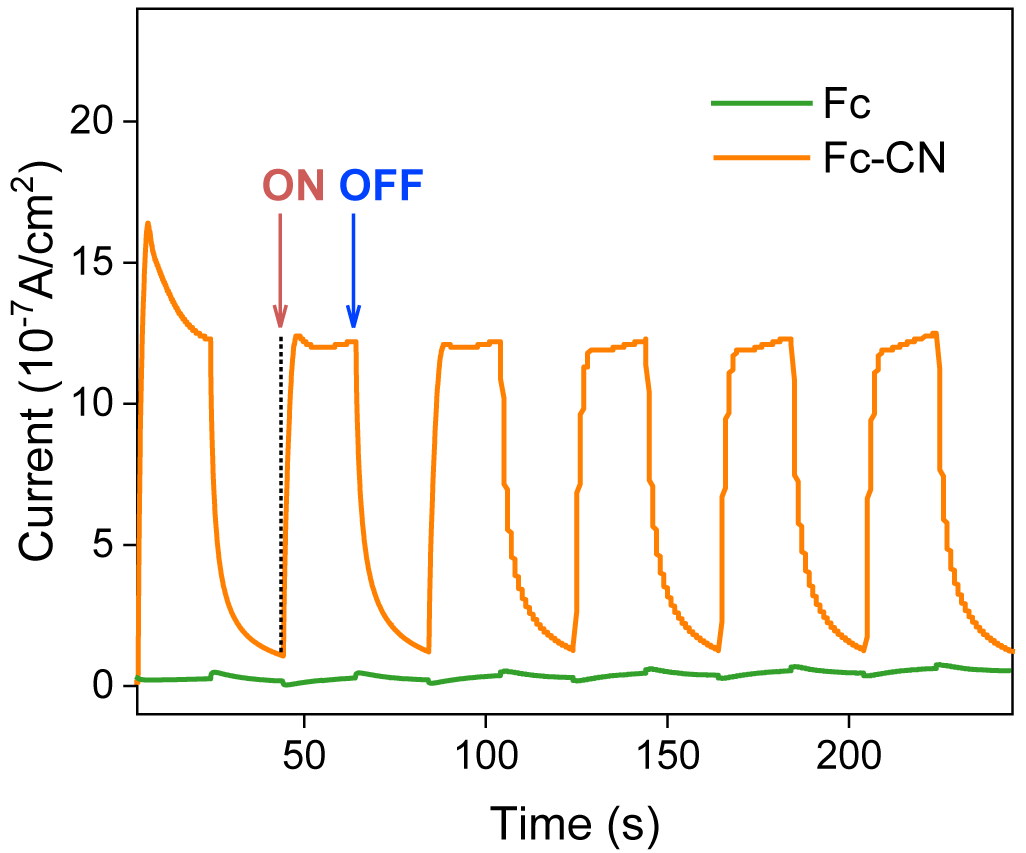


Figure S31. Transient photocurrent responses of Fc and Fc-CN. The transient photocurrent intensity analysis shows that Fc-CN exhibits obvious photocurrent, which is almost absent for Fc.


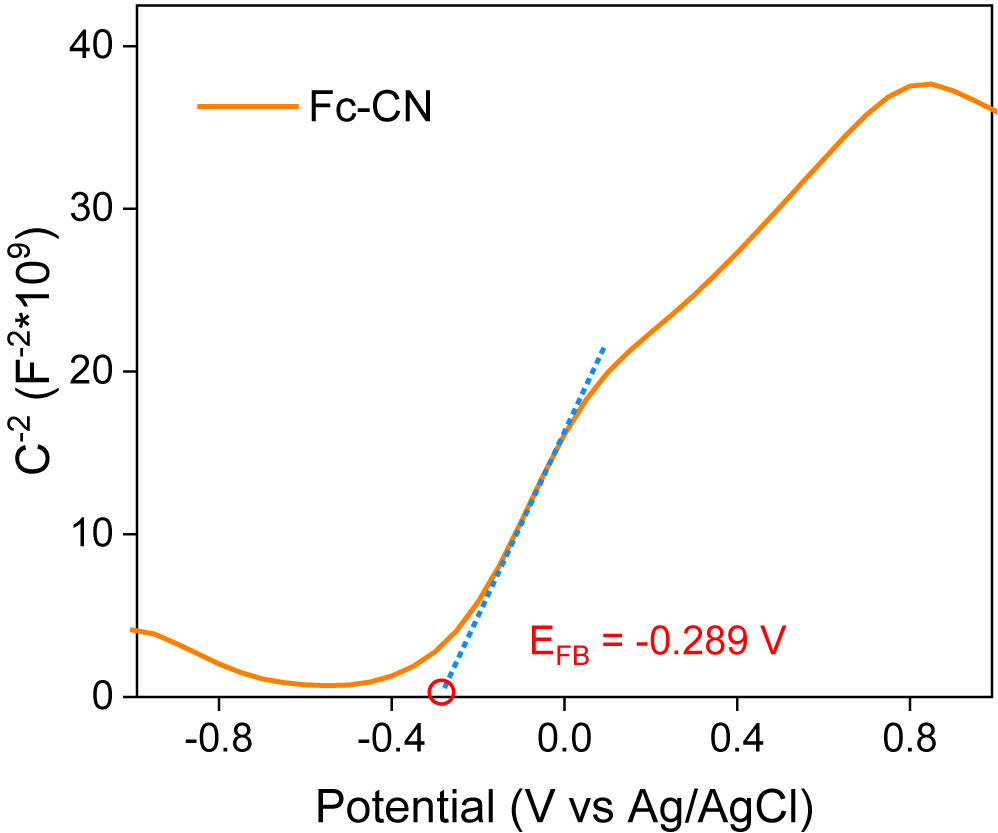


Figure S32. Mott-Schottky plot for Fc-CN nanocrystal.


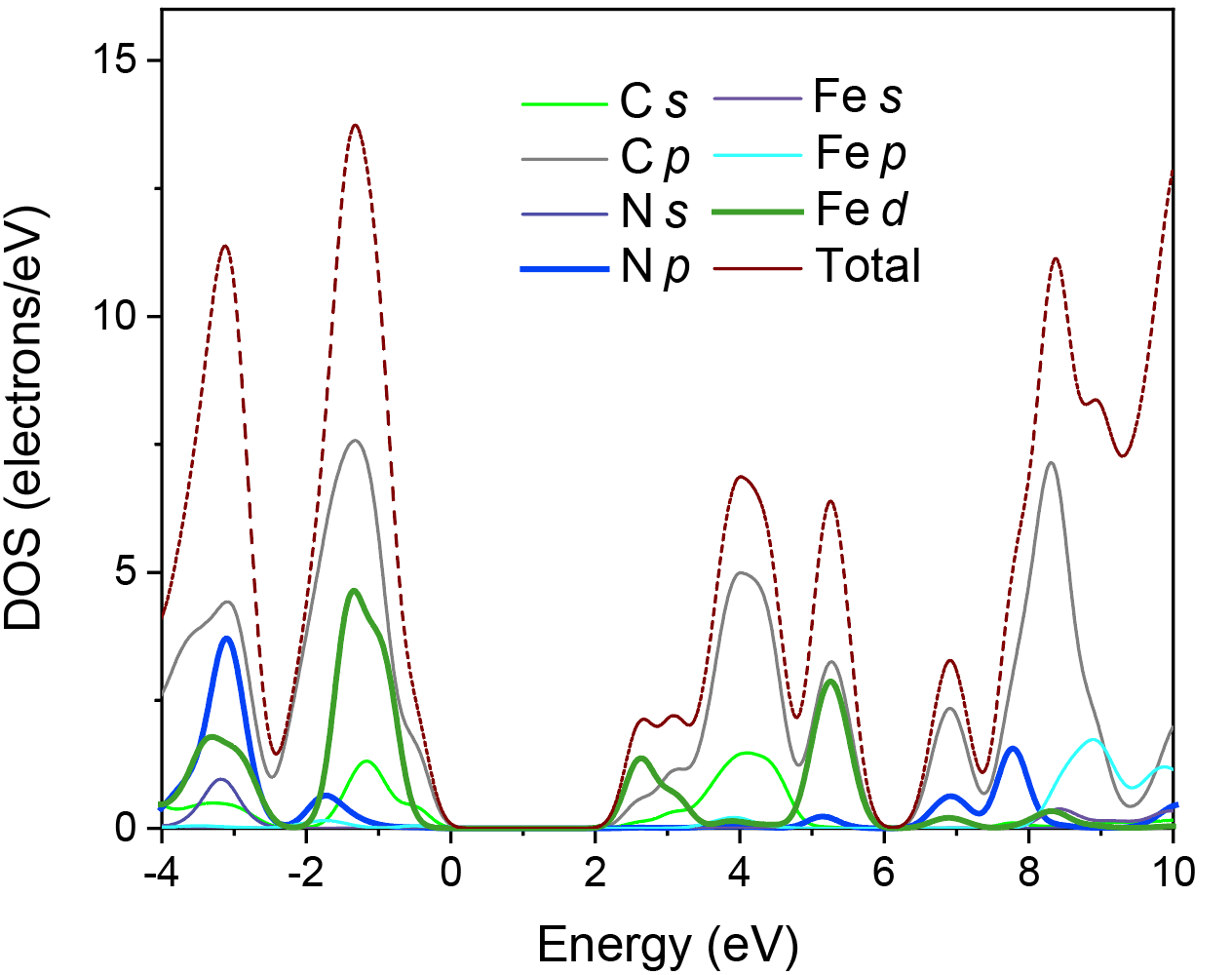


Figure S33. DOS analysis of Fc-CN molecule.


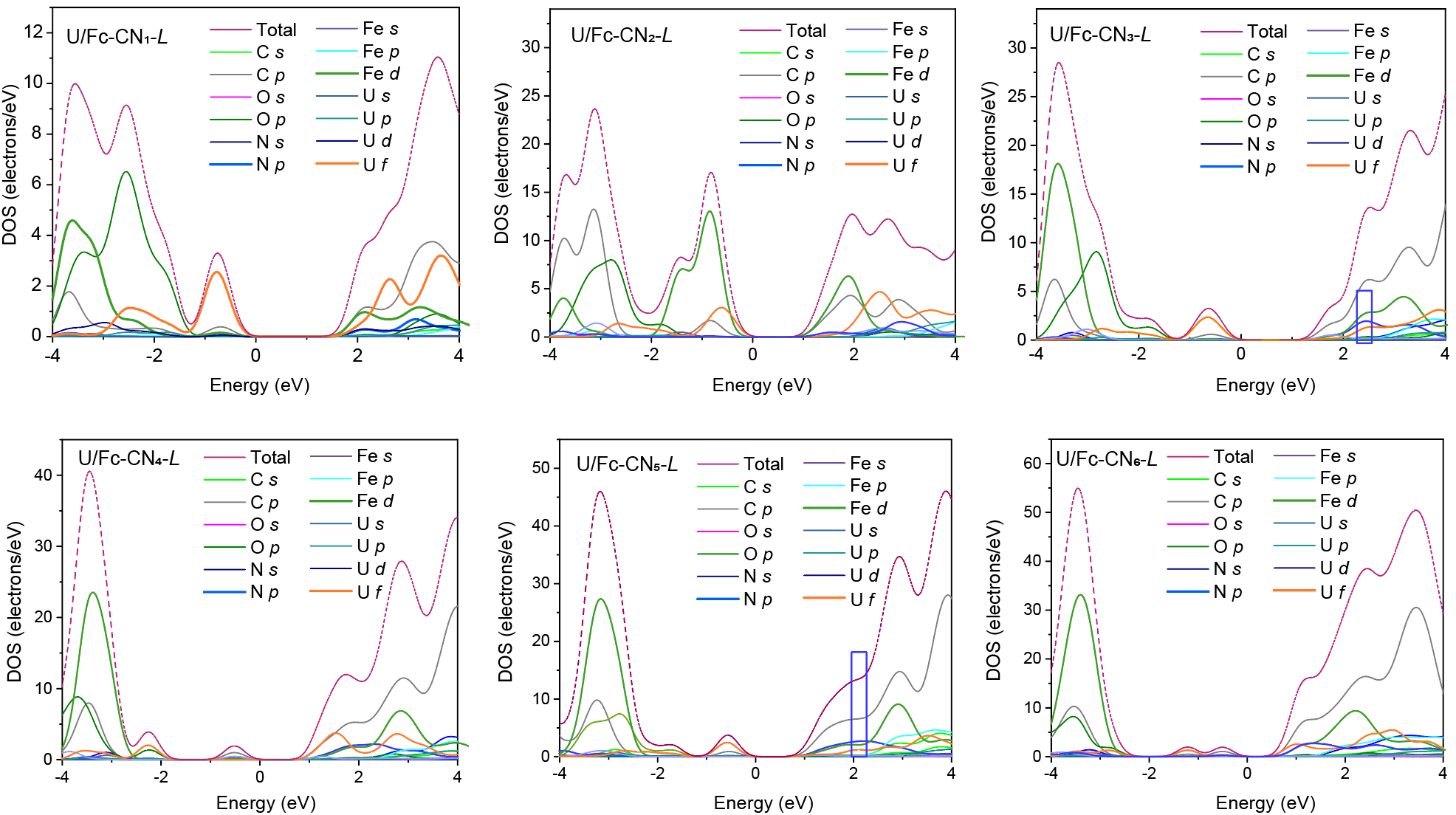


Figure S34. DOS analysis of U/Fc-CN_n_-*L* with different ratio of Fc-CN molecules and uranyl ions.


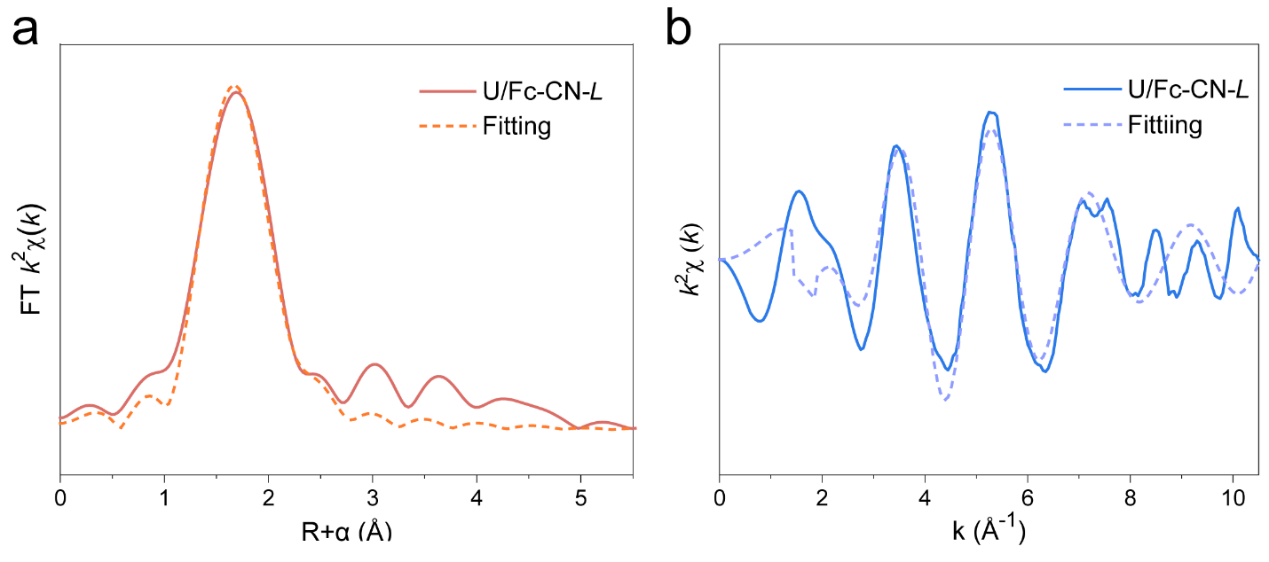


Figure S35. a) Fourier transform of the U *L_III_*-edge EXAFS spectra of U/Fc-CN-*L* in *R*-space. b) Accompanying *k^2^*-weighted *χ(k)* data and fit for U/Fc-CN-*L*.


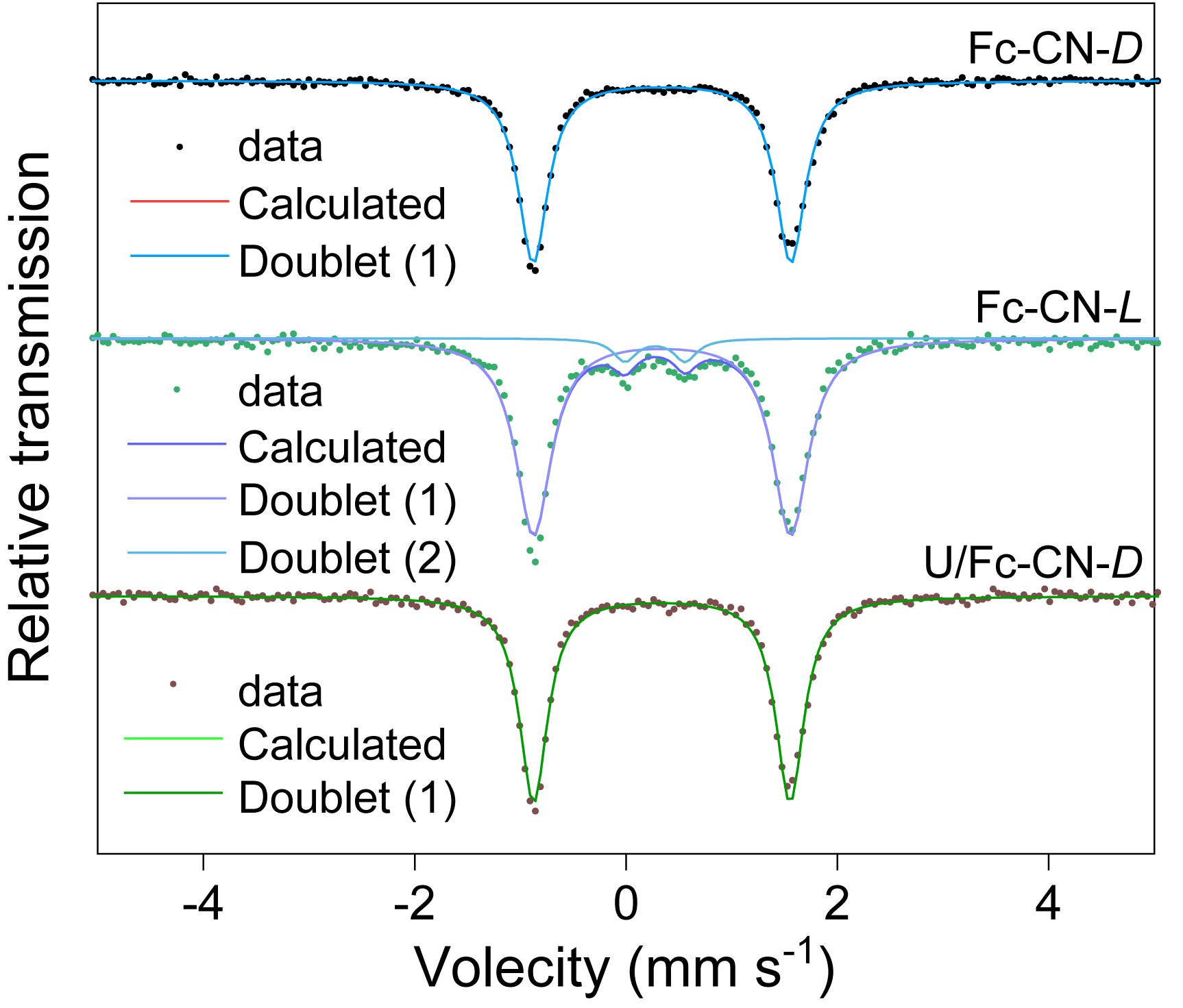


Figure S36. Room-temperature ^57^Fe Mössbauer spectra of Fc-CN-*D*, Fc-CN-*L*, and U/Fc-CN-*D*.


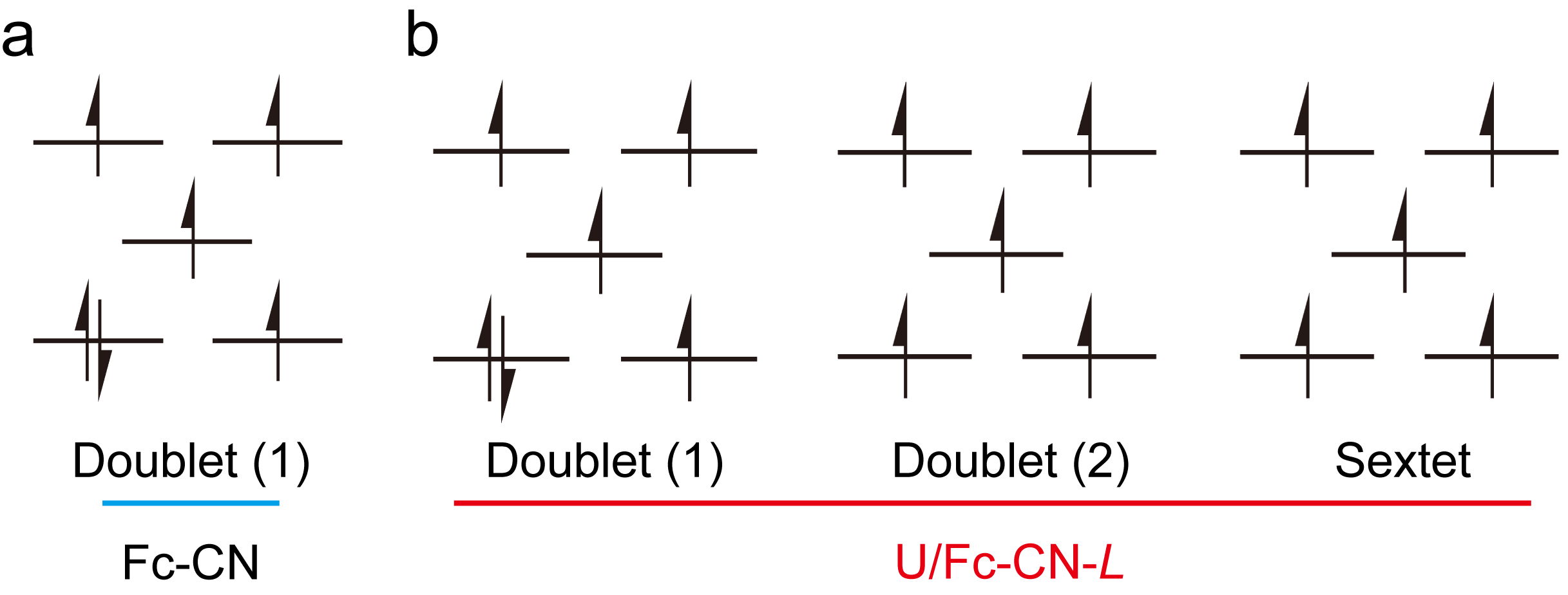


Figure S37. a) Molecular orbital diagram of Fe element in Fc-CN. b) Molecular orbital diagrams of Fe element in U/Fc-CN-*L*. The Fe element in Fc-CN has four unpaired spin electrons and a total of six spin electrons. After the formation of U/Fc-CN-*L*, the majority of Fe elements possess five unpaired spin electrons.

Table S1. Element composition of recrystallized Fc-CN obtained by organic elementals analyzers.

| Elements | C | H | N | Fe |
| --- | --- | --- | --- | --- |
| Contents (%) | 62.43 | 4.26 | 6.63 | 26.5 |

Table S2. The crystallographic data of recrystallized Fc-CN nanocrystal.

| / | Fc-CN |
| --- | --- |
| Empirical formula | C11H9FeN |
| Formula weight | 211.04 |
| Crystal system | Orthorhombic |
| Space group | p212121 |
| Unit cell dimensions | / |
| a | 7.5517(4) Å |
| b | 10.3749(7) Å |
| c | 11.0872(7) Å |
| a | 90° |
| b | 90° |
| g | 90° |
| Volume | 868 |
| Z | 4 |
| F (000) | 432 |
| Crystal size | 0.24×0.22×0.2 mm^3^ |
| Reflections collected | 10340 |
| Independent reflections | 1760 [R(int) = 0.0686] |
| Data/restraints/parameters | 1760/0/118 |
| Goodness-of-fit on F^2^ | 0.801 |

Table S3. The parameters of the pseudo-first-order and pseudo-second-order kinetic models for fitting the uranium separation kinetics of Fc-CN.

| Uranium concentration | Pseudo-first-order fitting model | Pseudo-second-order fitting model |
| --- | --- | --- |
|  | *R^2^* | *R^2^* |
| 8 ppm | 0.992 | 0.963 |
| 16 ppm | 0.939 | 0.955 |
| 32 ppm | 0.869 | 0.930 |

Table S4. Concentrations of metal ions in simulated nuclear wastewater used in this study.

| Metal ions | Concentrations of metal ions (ppm) | Metal ions | Concentrations of metal ions (ppm) |
| --- | --- | --- | --- |
| U(VI) | 18.9 | Zn(II) | 3.34 |
| Pb(II) | 10.1 | Co(II) | 3.08 |
| Ba(II) | 6.18 | Mg(II) | 1.53 |
| Cu(II) | 4.18 | Sr(II) | 4.22 |
| Ni(II) | 3.30 | Eu(III) | 7.1 |

Table S5. The adsorption capacity of adsorbents for uranium separation from nuclear wastewater.

| Adsorbents | Dosage  (g L^−1^) | Equilibration time  (min) | Adsorption capacity (mg g^−1^) | C_0_  (ppm) | Adsorption rate  (mg g^−1^ min^−1^) | Ref. |
| --- | --- | --- | --- | --- | --- | --- |
| Fc-CN | 0.1 | 70 | 185.18 | 18.9 | 2.645 | This work |
| C_3_N_5_/GO heterojunction | 0.5 | 90 | 19.22 | 10 | 0.213 | [1] |
| Ag-C-AO-wood | 10 | 1080 | 7.5 | 100 | 0.006 | [2] |
| MBTA-GO | 0.25 | 1440 | 63 | 119 | 0.043 | [3] |
| CC/PANI-2 | / | 120 | 216.48 | 100 | 1.804 | [4] |
| IIP-BSG | 1 | 90 | 149 | 187 | 1.655 | [5] |
| ZIF-8/PAN | 0.6 | 120 | 196 | 100 | 1.633 | [6] |
| Fe_3_O_4_@DFNS-NH_2_ | 0.4 | 120 | 112.5 | 50 | 0.078 | [7] |
| SPI hydrogel | 0.1 | 1440 | 53.94 | 18.9 | 0.037 | [8] |
| PAN-PC | 0.02 | 1440 | 433.5 | 20 | 0.301 | [9] |
| NCFs-HTO-BT | 0.4 | 1440 | 33 | 20 | 0.022 | [10] |
| M-AO-Fc | 0.08 | 60 | 84.31 | 18.9 | 1.405 | [11] |
| VPA@POSS | 0.333 | 120 | 273.2733 | 100 | 2.277 | [12] |
| PEPA/PDA-CMCS | 1 | 60 | 12.349 | 12.35 | 0.020 | [13] |
| AL-PEI/GMS | 1 | 90 | 19.98 | 20 | 0.222 | [14] |
| Solid-liquid / liquid-phase electrochemical/solid-phase recycling | / | 2160 | 98.9% | 199.92 | / | [15] |
| Porous biochar | 1 | 300 | 61.53 | 50 | 0.250 | [16] |
| PEI/ECH-CTS | 1 | 720 | 10.979 | 10.98 | 0.015 | [17] |
| HTO/CFs | 1 | 1440 | 23.2407 | 24.99 | 0.016 | [18] |

Table S6. Comparison of the adsorption capacity of Fc-CN nanocrystals and Fe_3_O_4_-based materials for uranium in nuclear wastewater.

| Adsorbents | Dosage  (g L^−1^) | Equilibration time  (min) | Adsorption capacity (mg g^−1^) | C_0_  (ppm) | Adsorption rate  (mg g^−1^ min^−1^) | Ref. |
| --- | --- | --- | --- | --- | --- | --- |
| Fc-CN | 0.1 | 70 | 185.18 | 18.9 | 2.645 | This work |
| Fe_3_O_4_@DFNS-NH_2_ | 0.4 | 120 | 112.5 | 50 | 0.078 | [7] |
| KMnFC/MA/Fe_3_O_4_ | 1 | 360 | 0.71 | 0.747 | 0.0019 | [19] |
| Fe_3_O_4_/β-CD/FA | 0.2 | 720 | 25.15 | 10 | 0.034 | [20] |
| Fe_3_O_4_@COFs | 0.1 | 30 | 9.88 | 1 | 0.329 | [21] |
| TiO_2_/PDA@Fe_3_O_4_ | 0.25 | 100 | 7.84 | 2 | 0.0784 | [22] |
| He-HNTs | 0.5 | 120 | 16.6 | 10 | 0.138 | [23] |
| Fe_3_O_4_@PAM | 0.25 | 1440 | 103.66 | 47.6 | 0.071 | [24] |
| h-Fe_3_O_4_@phos-PDA | 1.6 | 720 | 31.5 | 60 | 0.043 | [25] |
| Fe_3_O_4_/CNT | 0.2 | 360 | 68.17% | 120 | / | [26] |
| magnetite crystal | 0.2 | 120 | 45 | 10 | 0.375 | [27] |
| Fe_3_O_4_/gamma-Fe_2_O_3_ | 0.5 | 360 | 85.8 | 52 | 0.238 | [28] |
| Fe_3_O_4_@Ti_3_C_2_-PDA/OA | 0.2 | 1440 | 83 | 20 | 0.057 | [29] |
| Fe_3_O_4_/MWCNT | 0.6 | 1440 | 2.74 | 2.99 | 0.001 | [30] |
| APF | 1 | 180 | 13.87 | 16.4 | 0.077 | [31] |
| Fe_3_O_4_@SiO_2_-AO | 0.4 | 1440 | 112.6 | 50 | 0.078 | [32] |
| FTC | 0.1 | 1440 | 170.6 | 20 | 0.118 | [33] |

Table S7. U *L_III_*-edge EXAFS analysis of uranium-bearing U/Fc-CN-*L*.

| U/Fc-CN-*L* | shell | CN | R(Å) | σ^2^ |
| --- | --- | --- | --- | --- |
|  | U-O | 2.0 ± 0.4 | 1.97 ± 0.03 | 0.0201 |
|  | U-O | 3.9 ± 0.5 | 2.21 ± 0.02 | 0.0038 |
|  | U-N | 1.9 ± 0.4 | 2.39 ± 0.03 | 0.0053 |

CN: coordination numbers; *R*: bond distance; σ^2^: Debye-Waller factors.

Table S8. ^57^Fe Mössbauer spectra parameters of Fc-CN nanocrystals after different treatments.

| Sample | Component | IS (mm/s) | QS (mm/s) | H (T) | Area (%) |
| --- | --- | --- | --- | --- | --- |
| Fc-CN | Doublet (1) | 0.34 | 2.42 | / | 100 |
| Fc-CN-*D* | Doublet (1) | 0.34 | 2.44 | / | 100 |
| Fc-CN-*L* | Doublet (1) | 0.34 | 2.43 | / | 93.0 |
|  | Doublet (2) | 0.27 | 0.57 | / | 7 |
| U/Fc-CN-*D* | Doublet (1) | 0.34 | 2.43 | / | 100 |
| U/Fc-CN-*L* | Doublet (1) | 0.37 | 2.29 | / | 6.3 |
|  | Doublet (2) | 0.27 | 0.63 | / | 52.0 |
|  | Sextet (1) | 0.24 | 0.00 | 49.15 | 41.7 |

IS: isomer shifts, QS: quadrupole splitting, and H: magnetic hyperfine filed.

References

[1] Q. Meng, X. Yang, L. Wu, T. Chen, Y. Li, R. He, W. Zhu, L. Zhu, T. Duan, *J. Hazard. Mater.* 2022, *422*, 126912.

[2] Z. Wang, Z. R. Wang, Z. X. Jiang, Y. He, T. Duan, *Environ. Sci. Pollut. Res.* 2021, *28*, 46053−46062.

[3] J. Ding, Z. J. Yan, L. Q. Feng, F. W. Zhai, X. Chen, Y. W. Xu, S. Q. Tang, C. Huang, L. C. Li, N. Pan, Y. He, Y. D. Jin, C. Q. Xia, *Environ. Pollut.* 2019, *253*, 221−230.

[4] H. Yu, L. Zhou, Y. Liu, X. Ao, J. Ouyang, Z. Liu, A. A. Adesina, *Desalination* 2023, *564*, 116773.

[5] Y. Su, M. Wenzel, M. Seifert, J. J. Weigand, *J. Hazard. Mater.* 2022, *440*, 129682.

[6] C. Wang, T. Zheng, R. Luo, C. Liu, M. Zhang, J. Li, X. Sun, J. Shen, W. Han, L. Wang, *ACS Appl. Mater. Interfaces* 2018, *10*, 24164−24171.

[7] F. Wang, T. Li, Y. Liao, L. Xia, *Appl. Surf. Sci.* 2023, *638*, 157969.

[8] M. Cao, Q. Peng, Y. Wang, G. S. Luo, L. J. Feng, S. L. Zhao, Y. H. Yuan, N. Wang, *Int. J. Biol. Macromol.* 2023, *242*, 124792.

[9] H. Li, L. Y. Li, J. Wen, G. Ye, J. Chen, X. L. Wang, *Chem. Eng. J.* 2023, *456*, 140935.

[10] J. Yu, C. Yu, W. Zhu, G. He, Y. Wei, J. Zhou, *Chemosphere* 2022, *286*, 131626.

[11] S. Zhao, T. Feng, L. Feng, B. Yan, W. Sun, G. Luo, M. Wang, Y. Jian, T. Liu, Y. Yuan, N. Wang, *Sep. Purif. Technol.* 2022, *287*, 120524.

[12] X. Zhang, C. Luo, H. Li, R. Liu, H. Wang, L. Liu, C. Yue, *React. Funct. Polym.* 2023, *190*, 105648.

[13] M. Huang, S. Fan, L. Xie, J. Cui, W. Shan, Z. Lou, Y. Xiong, *Sep. Purif. Technol.* 2023, *321*, 124198.

[14] L. Guo, L. Peng, J. Li, W. Zhang, B. Shi, *J. Clean. Prod.* 2023, *388*, 136006.

[15] J. Gao, J. Chen, H. Lv, S. Liao, Y. Yan, Y. Xue, F. Ma, S. Wang, *Chem. Eng. J.* 2023, *460*, 141784.

[16] Y. Liu, W. Yuan, W. Lin, S. Yu, L. Zhou, Q. Zeng, J. Wang, L. Tao, Q. Dai, J. Liu, *Environ. Pollut.* 2023, *335*, 122262.

[17] M. Huang, L. Xie, Y. Wang, X. Feng, J. Gao, Z. Lou, Y. Xiong, *Environ. Pollut.* 2023, *316*, 120550.

[18] Y. Tang, J. Zhou, J. Guo, X. Liao, B. Shi, *J. Environ. Manage.* 2021, *283*, 112001.

[19] T. T. Li, F. Wang, L. S. Xia, *Radiochim. Acta* 2024, *112*, 301.

[20] Y. Zhang, S. Q. Huang, B. Y. Mei, X. Y. Tian, L. Y. Jia, N. Sun, *Chemosphere* 2023, *331*.

[21] S. Yang, Z. Y. Ye, X. Y. Cheng, Y. C. Wang, Z. T. Luan, W. J. Li, B. W. Hu, *Chem. Eng. J.* 2023, *474*.

[22] P. Zhang, H. Li, Y. H. Wang, J. F. Song, J. Huang, P. Li, *J. Clean. Prod.* 2023, *425*.

[23] M. H. Su, T. Ou, J. Y. Li, L. Z. Tong, W. X. Han, Y. H. Wu, D. Y. Chen, *J. Environ. Chem. Eng.* 2023, *11*.

[24] W. C. Song, M. C. Liu, R. Hu, X. L. Tan, J. X. Li, *Chem. Eng. J.* 2014, *246*, 268.

[25] J. F. Zhang, D. Wang, R. Y. Cao, F. W. Sun, J. X. Li, *J. Environ. Chem. Eng.* 2022, *10*.

[26] J. F. Zhang, Y. H. Wang, Y. J. Wei, M. Xu, Y. H. Hu, J. X. Li, *J. Environ. Chem. Eng.* 2024, *12*.

[27] D. M. Fu, M. Li, Y. L. Hua, F. Y. Gao, X. Y. Wu, X. W. Zhang, Q. Fang, L. Bi, T. Cai, *Sep. Purif. Technol.* 2022, *291*.

[28] Y. L. Hua, W. Wang, X. Y. Huang, T. H. Gu, D. X. Ding, L. Ling, W. X. Zhang, *Chemosphere* 2018, *201*, 603.

[29] F. L. Liu, Z. F. Hu, M. Xiang, B. W. Hu, *Appl. Surf. Sci.* 2022, *601*.

[30] P. F. Zong, J. Y. Gou, *J. Mol. Liq.* 2014, *195*, 92.

[31] J. Y. Li, W. X. Han, H. Y. Liu, M. H. Su, D. Y. Chen, G. Song, *J. Clean. Prod.* 2023, *409*.

[32] J. M. Liu, X. H. Yin, T. Liu, *J. Taiwan Inst. Chem. Eng.* 2019, *95*, 416.

[33] C. Wang, H. Jiao, Y. B. Yang, Y. C. Wu, P. Na, *J. Clean. Prod.* 2023, *426*.
